# The mTORC1-4E-BP-eIF4E axis controls de novo Bcl6 protein synthesis in T cells and systemic autoimmunity

**DOI:** 10.1038/s41467-017-00348-3

**Published:** 2017-08-15

**Authors:** Woelsung Yi, Sanjay Gupta, Edd Ricker, Michela Manni, Rolf Jessberger, Yurii Chinenov, Henrik Molina, Alessandra B. Pernis

**Affiliations:** 1 Autoimmunity and Inflammation Program, Hospital for Special Surgery, 535 East 70th Street, New York, New York 10021 USA; 2 Graduate Program in Immunology and Microbial Pathogenesis, Weill Cornell Graduate School of Medical Sciences, 1300 York Avenue, Box 65, New York, New York 10021 USA; 3 Institute of Physiological Chemistry, Technische Universität Dresden, Fiedlerstrasse 42, MTZ, 01307 Dresden, Germany; 4 Arthritis and Tissue Degeneration Program, Hospital for Special Surgery, 535 East 70th Street, New York, New York 10021 USA; 5 David Z. Rosensweig Genomics Research Center, Hospital for Special Surgery, 535 East 70th Street, New York, New York 10021 USA; 6 Proteomics Resource Center, The Rockefeller University, 1230 York Avenue, Box 105, New York, New York 10065 USA; 7 Department of Medicine, Weill Cornell Medical College, Cornell University, 525 East 68th Street, Box 130, New York, New York 10065 USA

## Abstract

Post-transcriptional modifications can control protein abundance, but the extent to which these alterations contribute to the expression of T helper (T_H_) lineage-defining factors is unknown. Tight regulation of Bcl6 expression, an essential transcription factor for T follicular helper (T_FH_) cells, is critical as aberrant T_FH_ cell expansion is associated with autoimmune diseases, such as systemic lupus erythematosus (SLE). Here we show that lack of the SLE risk variant *Def6* results in deregulation of Bcl6 protein synthesis in T cells as a result of enhanced activation of the mTORC1–4E-BP–eIF4E axis, secondary to aberrant assembly of a raptor–p62–TRAF6 complex. Proteomic analysis reveals that this pathway selectively controls the abundance of a subset of proteins. Rapamycin or raptor deletion ameliorates the aberrant T_FH_ cell expansion in mice lacking Def6. Thus deregulation of mTORC1-dependent pathways controlling protein synthesis can result in T-cell dysfunction, indicating a mechanism by which mTORC1 can promote autoimmunity.

## Abstract (web summary)

Excessive expansion of the T follicular helper (T_FH_) cell pool is associated with autoimmune disease and *Def6* has been identified as an SLE risk variant. Here the authors show that Def6 limits proliferation of T_FH_ cells in mice via alteration of mTORC1 signaling and inhibition of Bcl6 expression.

## Introduction

Precise regulation of T follicular helper (T_FH_) cell numbers is critical for optimal humoral responses, and aberrant expansion of T_FH_ cells is associated with autoimmune diseases, including systemic lupus erythematosus (SLE)^1, 2^. The transcriptional repressor Bcl6 is a lineage-defining factor for T_FH_ cells^3–5^. Bcl6 is necessary to specify the T_FH_ cell program and overexpression of Bcl6 is sufficient to drive T_FH_ cell differentiation, indicating that tight control of Bcl6 expression is essential to ensure proper regulation of T_FH_ cell numbers. Bcl6 expression in T_FH_ cells has, until now, been shown to be primarily regulated by transcriptional mechanisms^6^. The expression of Bcl6, however, can be controlled by complex regulatory networks that fine-tune Bcl6 expression by targeting both *Bcl6* mRNA and protein^7^. In B cells, Bcl6 levels are regulated by a number of post-transcriptional mechanisms, which control Bcl6 protein stability and its activity^7^.

Among post-transcriptional mechanisms, translational control has a major function in regulating protein abundance and can influence protein levels to an extent similar to transcription^8^. A critical controller of protein synthesis is mammalian target of rapamycin (mTOR), a serine/threonine kinase that exists in two distinct complexes, mTORC1 and mTORC2, distinguished by the presence of unique components such as raptor and rictor, respectively^9, 10^. mTORC1 activation occurs in response to diverse environmental cues, including growth factors, energy status, and amino-acid availability. Growth factors activate mTORC1 mainly through the phosphoinositide-3 kinase (PI3K)-AKT pathway, whereas the energy status of a cell regulates mTORC1 activation via AMP-activated protein kinase (AMPK)^9–11^. mTORC1 activation by PI3K-AKT and AMPK occurs via the TSC complex and the small GTPAse Rheb^9–11^. By contrast, amino acids regulate a different set of GTPases, the Rag proteins, which recruit mTORC1 to the lysosomes enabling subsequent activation by Rheb. Although activation of the Rags normally depends on their interaction with the Ragulator complex, an alternative docking system that depends on the central signaling hub p62 can also control activation^11–13^. p62 interacts with and activates the Rags, helps recruit mTORC1 to the lysosomes by binding Raptor and also mediates the assembly of a trimolecular complex with TRAF6, which can then activate mTOR kinase activity via K63-linked polyubiquitination^12, 13^.

mTOR is a major coordinator of T_H_ cell fate decisions and regulates the differentiation of several T_H_ subsets^9, 10^. mTOR plays a complex role in T_FH_ differentiation. Whereas the interleukin (IL)-2–mTORC1 axis shifted differentiation away from T_FH_ cells toward the T_H_1 lineage in an acute viral infection model^14^, mTORC1 activation is required for the spontaneous formation of T_FH_ cells in Peyer’s patches and for the induction of T_FH_ cells upon immunization with a foreign antigen^15, 16^. mTORC2 activity is also important for T_FH_ differentiation, particularly in Peyer’s patches^16^. The varying requirements of T_FH_ cells on mTOR activity are probably due to differences in the precise environmental cues to which T_FH_ cells are exposed^16^. mTOR has been shown to regulate T_H_ cell differentiation by controlling the transcription of master regulators and metabolic reprogramming. Although regulation of protein synthesis is also a major downstream function of mTORC1, its role in T_H_ cells is less well understood.

mTOR has been implicated in the pathogenesis of autoimmune disorders, like SLE^17^. The pathways resulting in mTOR deregulation and T_H_ cell dysfunction in autoimmunity are, however, not fully understood. *Def6* is a an SLE risk variant^18^, which together with its only homolog SWAP-70, comprises the SWEF family of molecules^19^. Unlike SWAP-70, which is expressed by B cells but not naive T_H_ cells^20^, Def6 is highly expressed by naive T_H_ cells. Notably, double knockout (DKO) of *Def6* and *SWAP70* in C57BL/6 mice results in development of lupus, predominantly in female mice as in human SLE^21^. Autoimmunity in DKO mice results from dual abnormalities in T and B cells, whereby the lack of *Def6* alone is responsible for the T-cell abnormalities, and the absence of both *Def6* and *SWAP70* contributes to the deregulated B-cell responses^21^.

In this study, we demonstrate that the robust humoral autoimmune responses observed in *Def6* ^*tr/tr*^ *Swap70* ^*−/−*^ DKO mice are accompanied by cell-intrinsic expansion of the T_FH_ cell compartment. Importantly, we show that DKO T cells have aberrant control of Bcl6 protein synthesis, which occurs in an mTORC1 and eukaryotic initiation factor 4E (eIF4E)-dependent manner. Enhanced mTORC1 activation in DKO T cells is a result of dysregulated interaction of raptor with both p62 and TRAF6, critical regulators of an amino-acid-sensing pathway of mTORC1 activation^12, 13^. We also demonstrate that Def6 controls the assembly of the raptor–p62–TRAF6 complex and that this pathway selectively regulates the abundance of a specific subset of proteins. Consistent with these findings, rapamycin administration or T-cell deletion of raptor significantly decreased the accumulation of T_FH_ cells in DKO mice. Thus abnormalities in the mechanisms by which mTORC1 regulates protein abundance can result in T-cell dysfunction and contribute to autoimmunity.

## Results

### Spontaneous expansion of T_FH_ cells in lupus-prone DKO mice

**Fig. 1:**
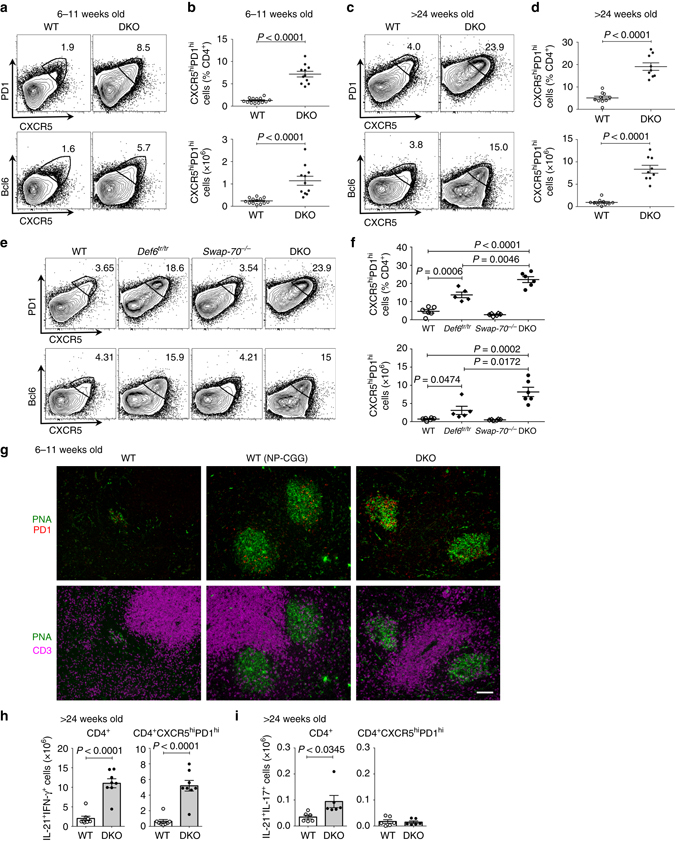
Spontaneous expansion of T_FH_ cells in DKO mice. Flow cytometric analysis of T_FH_ cells in the spleens from young (6–11-week old) **a**, **b** and aging (>24-week old) **c**, **d** wt or DKO (*Def6* ^*tr/tr*^ *Swap70* ^*−/−*^) mice. **a**, **c** Representative FACS plots for CXCR5 and PD1 (*upper* panel) or CXCR5 and Bcl6 (*lower* panel) expression gated on CD4^+^ T cells. **b**, **d** Quantification of the frequencies and numbers of T_FH_ cells (CD4^+^CXCR5^hi^PD1^hi^) (*n* = 10–13). **e**, **f** Flow cytometric analysis of T_FH_ cells in wt, *Def6* ^*tr/tr*^, *Swap70* ^*−/−*^, and DKO mice. **e** Representative FACS plots for CXCR5 and PD1 (*upper* panel) or CXCR5 and Bcl6 (*lower* panel) expression gated on CD4^+^ T cells in the spleens from mice of the indicated genotype (>18 weeks). **f** Quantification of T_FH_ cells (CD4^+^CXCR5^hi^PD1^hi^) (*n* = 5–7). Combined data from three independent experiments. **g** Representative immunofluorescence images of splenic sections from wt unimmunized mice, wt mice 8 days post-immunization with NP-CGG and DKO unimmunized mice (two independent experiments, *n* = 2–3 each). PNA, *green*; PD1, *red*; CD3, *magenta*; *Scale bar*, 100 μm. **h**, **i** Quantification of CD4^+^ T cells producing IL-17, IL-21, and IFN-γ in the spleens of aging wt and DKO mice (*n* = 4–8) analyzed by intracellular flow cytometry. Each dot represents an individual mouse. *Error bars* indicate mean ± s.d., *P*-value by two-tailed *t*-test. Data were combined from at least two independent experiments

Precise control of T_FH_ cell differentiation is essential to prevent SLE^1, 2^. Mice lacking *Def6* and *Swap70* (here termed DKO mice) spontaneously develop a systemic autoimmune disorder characterized by increased numbers of effector T cells, germinal center (GC) B cells, plasma cells, and autoantibody production^21^. This robust humoral autoimmune response led us to speculate that many of the effector T cells that accumulate in these mice might be T_FH_ cells. A detailed analysis indeed revealed that young DKO mice spontaneously display increased frequencies and numbers of T_FH_ cells based on staining for either CXCR5^hi^PD1^hi^ CD4^+^ T cells or CXCR5^hi^Bcl6^hi^ CD4^+^ T cells (Figs. 1a, b). Similar results were also obtained when T_FR_ cells, a subset of CXCR5^hi^PD1^hi^ cells that expresses Foxp3 and specialize in the inhibition of the GC reaction^22, 23^, was excluded from the analysis by gating out Foxp3^+^ cells (Supplementary Figs. 1a, b, e, f). DKO T_FH_ cells further expanded with age (Figs. 1c, d and Supplementary Fig. 1c) and their accumulation correlated with increased frequencies of GC B cells and plasma cells (Supplementary Figs. 2a–h). Analysis of mice deficient in either *Def6* alone or *Swap70* alone demonstrated that the lack of Def6 is largely responsible for the increase in T_FH_ cells in DKO mice, consistent with the finding that naive T helper cells express Def6 but not SWAP-70 (Figs. 1e–f). Thus T_FH_ cells spontaneously accumulate in lupus-prone DKO mice.

To establish whether DKO T_FH_ cells were located within the spontaneous GCs that develop in these mice, we stained splenic sections from unimmunized or immunized wild-type (wt) mice or unimmunized DKO mice with PNA and PD1, markers previously utilized to identify GC B cells and T_FH_ cells, respectively^24^. Few PD1^+^ cells were detected in unimmunized wt mice while PD1^+^ cells could easily be visualized within the PNA^+^ GCs of immunized wt mice (Fig. 1g, *top* panel). Costaining of PD1^+^ cells with CD3 confirmed their T-cell identity (Fig. 1g, *bottom* panel). Similarly to immunized wt mice, young unimmunized DKO mice contained well-formed PNA^+^ GCs with PD1^+^ T cells, which appeared to be diffusely distributed throughout the GCs (Fig. 1g and Supplementary Fig. 3a)^25^. A similar localization pattern was also observed in aging DKO female mice (Supplementary Fig. 3b). T_FH_ cells in DKO mice thus accumulate within GCs although some PD1^+^ T cells could also be observed at extrafollicular sites.

T_FH_ cells produce various cytokines that have been implicated in autoimmune pathogenesis including interferon (IFN)-γ and IL-21^1, 2^. Excessive IFN-γ production has been associated with the accumulation of pathogenic T_FH_ cells in sanroque mice^26^. Evaluation of the cytokine profile of DKO T_FH_ cells revealed an expansion of T_FH_ cells that produce both IFN-γ and IL-21 (Fig. 1h and Supplementary Figs. 3c, d). While DKO mice also exhibited an increased number of CD4^+^ T cells producing both IL-17 and IL-21, this effect was primarily restricted to non-T_FH_ effector T cells (Fig. 1i and Supplementary Fig. 3e). The T_FH_ cells that accumulate in DKO mice are thus capable of producing cytokines that have been linked to the development of systemic autoimmunity.

**Fig. 2:**
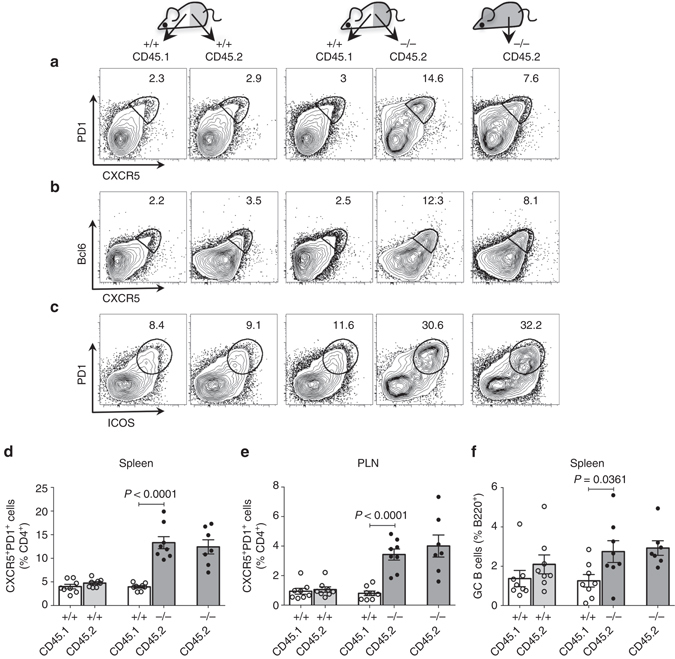
Expansion of T_FH_ cells in DKO mice is cell-intrinsic. Analysis of mixed bone marrow chimeric mice (*n* = 8) 8–10 weeks after the reconstitution in *Rag-1*-deficient hosts. Chimeric mice were generated with 1:1 ratio of wild-type CD45.1 and wild-type CD45.2 bone marrow cells (control) or wild-type CD45.1^+^ and DKO CD45.2^+^ bone marrow cells (experimental) or only DKO CD45.2^+^ bone marrow cells (reference). **a**–**c** FACS plots for CXCR5 and PD1 **a** or CXCR5 and Bcl6 **b** or ICOS and PD1 **c** expression gated on CD4^+^ T cells. **d**, **e** Frequencies of T_FH_ cells in the chimeric mice of the indicated genotypes. **f** Frequencies of GC B cells in the chimeric mice of the indicated genotypes. *PLN* peripheral lymph nodes. Each dot represents an individual mouse. *Error bars* indicate mean ± s.d., *P*-value by two-tailed *t*-test. Combined data from two independent experiments

To evaluate whether the spontaneous expansion of T_FH_ cells in DKO mice is cell-intrinsic, we generated mixed bone marrow chimeras. *Rag1* ^*−/−*^ mice reconstituted with only CD45.2^+^ DKO bone marrow cells were also generated as control. The recipient mice were then analyzed for the presence of CXCR5^hi^PD1^hi^ T_FH_ cells in the spleen and peripheral lymph nodes. CD45.2^+^ DKO T cells contained an increased proportion of T_FH_ cells, which reached levels comparable to those observed in mice reconstituted with CD45.2^+^ DKO bone marrow cells alone (Figs. 2a, d, e). Similar results were also obtained by staining for CXCR5^hi^Bcl6^hi^ or PD1^hi^ICOS^hi^ T cells (Figs. 2b, c). A slightly greater propensity of DKO B cells to acquire a GC B-cell phenotype as compared to wt B cells could also be observed (Fig. 2f). Collectively, these data support the notion that the spontaneous expansion of DKO T_FH_ cells is cell-autonomous.

### Aberrant control of Bcl6 protein synthesis in DKO T cells

**Fig. 3:**
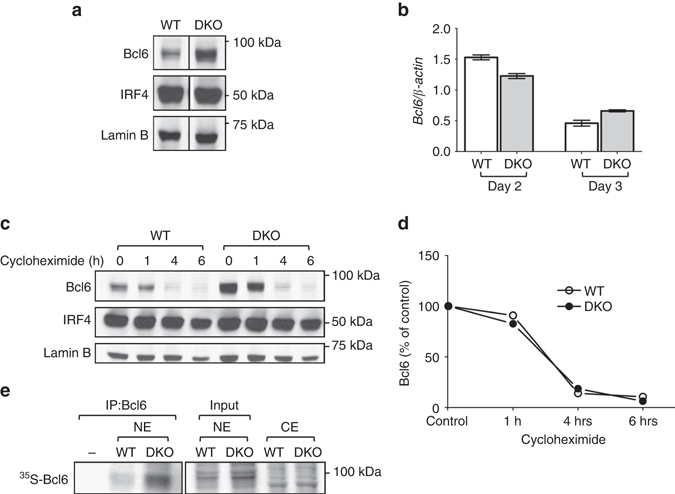
De novo protein synthesis of Bcl6 is increased in DKO T cells. **a** FACS-sorted naive T cells (CD4^+^CD25^−^CD44^−^CD62L^+^) from wt and DKO mice were stimulated with anti-CD3 and anti-CD28 for 3 days. Nuclear extracts were evaluated by western blotting with antibodies to Bcl6 and IRF4. Lamin B was used as a loading control. Data are representative of three independent experiments. **b** Quantitative RT–PCR analysis of the expression of *Bcl6* mRNA in sorted naive T cells cultured for 2 or 3 days as indicated. The data were normalized relative to *β-Actin* mRNA expression. Data are representative of two independent experiments. **c** Assessment of Bcl6 protein stability was conducted by exposing wt or DKO T cells cultured as in **a** to either vehicle control or cycloheximide (100 μg/ml) for the indicated times during the final hours of the 3-day culture. Nuclear extracts were assessed by western blotting. IRF4 and Lamin B were used as control. Data are representative of two independent experiments. **d** Quantification of Bcl6 signal intensity by densitometry over the time course of cycloheximide treatment. The levels of Bcl6 in the vehicle-treated controls were set as 100%. **e** Autoradiography of SDS-PAGE gel to detect [^35^S]-labeled Bcl6 protein immunoprecipitated from nuclear extracts of wt or DKO T cells cultured as in **a** followed by metabolic labeling with [^35^S]methionine/cysteine for 1 h. Data are representative of two independent experiments. *NE* nuclear extract, *CE* cytoplasmic extract. *Error bars* indicate mean ± s.d., *P*-value by two-tailed *t*-test

The spontaneous cell-intrinsic expansion of pathogenic T_FH_ cells in the lupus-prone DKO mice suggested that DKO T cells inappropriately acquire features of T_FH_ cells. In view of the critical role of Bcl6 in the differentiation of T_FH_ cells^3–5^, we focused our attention on this transcription factor. To investigate whether Bcl6 expression might be dysregulated in DKO T cells, we sorted naive CD4^+^ T cells from wt and DKO mice, which expressed similar low levels of Bcl6 protein (Supplementary Fig. 4a), and activated them in vitro. Remarkably, as compared to wt T cells, DKO T cells exhibited much higher Bcl6 protein levels (Fig. 3a and Supplementary Fig. 4b). In line with the finding that Def6 is the primary member of this small family of proteins expressed in T cells^27, 28^, similar results were obtained in T cells lacking Def6 alone while T cells from SWAP-70-deficient mice were indistinguishable from wt T cells (Supplementary Fig. 4c). No phosphorylation of Stat1, Stat3 or Stat4 was detected in DKO T cells, suggesting that the high levels of Bcl6 expression were not due to inappropriate STAT activation or aberrant production of IL-6 or IL-21 (Supplementary Fig. 4d). Furthermore, the expression of interferon regulatory factor 4 (IRF4), which has been implicated in the differentiation of several T_H_ cell lineages including T_FH_ cells, was similar in wt and DKO T cells (Fig. 3a). Interestingly, we observed a similar increase in Bcl6 protein expression in DKO T cells cultured under T_H_1 conditions although T-bet levels were upregulated to a similar extent in wt and DKO T cells upon T_H_1 skewing (Supplementary Fig. 4e). Surprisingly, *Bcl6* mRNA was not upregulated in DKO T cells as compared to wt T cells as assessed by quantitative reverse transcription–PCR (RT–PCR) with two different sets of primers and at different time points (Fig. 3b and Supplementary Figs. 4f, g). Thus the expression of a key controller of T_FH_ cell differentiation, Bcl6, is selectively dysregulated in DKO T cells and this effect is not due to a transcriptional mechanism or to global alterations in the ability of DKO T cells to undergo T_H_ differentiation.

In B cells, post-transcriptional mechanisms play an important role in regulating Bcl6 protein levels^7^. In particular, Bcl6 contains three PEST domains and can be rapidly degraded. To evaluate whether the elevated levels of Bcl6 protein in DKO T cells could be due to alterations in the stability of the Bcl6 protein, wt or DKO T cells were treated with cycloheximide, a protein synthesis inhibitor, and the levels of Bcl6 protein monitored over time (Figs. 3c, d). In contrast to IRF4, which was very stable, Bcl6 protein was rapidly degraded following cycloheximide treatment. The rate of degradation of Bcl6, however, was similar between wt and DKO T cells (Figs. 3c, d), indicating that the accumulation of Bcl6 protein in DKO T cells is not secondary to changes in protein stability.

We next considered the possibility that Bcl6 protein accumulated in DKO T cells due to alterations in de novo Bcl6 protein synthesis. To evaluate this possibility, we performed metabolic labeling assays. Wt and DKO T cells were metabolically labeled with [^35^S]methionine and the incorporation of [^35^S]methionine in Bcl6 protein was assessed by Bcl6 immunoprecipitation followed by autoradiography. Higher levels of [^35^S]methionine incorporation were detected in Bcl6 immunoprecipitates from DKO T cells as compared to wt T cells (Fig. 3e). Interestingly, the [^35^S]methionine incorporation assays did not reveal a global dysregulation in protein synthesis in DKO T cells (Supplementary Fig. 4h). Thus the increased levels of Bcl6 protein expression in DKO T cells are due to aberrancies in the control mechanisms that regulate the de novo synthesis of Bcl6 protein.

### mTORC1 controls Bcl6 protein levels in DKO T cells

**Fig. 4:**
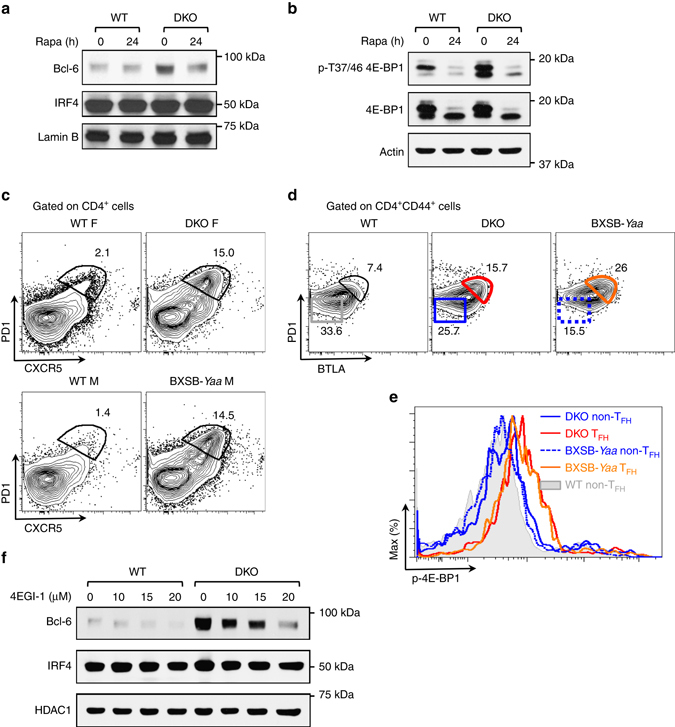
Expression of Bcl6 protein in DKO T cells is mTOR dependent. **a** Naive T cells from wt and DKO mice were cultured as described in Fig. 3 and treated with vehicle control or rapamycin (20 nM) for the final 24 h of the 3-day culture. Bcl6 expression was assessed by western blotting using nuclear extracts. Levels of IRF4 and Lamin B were evaluated as control. **b** Western blotting analysis of the levels of p-4E-BP in extracts from wt or DKO T cells. **c** Representative FACS plots for CXCR5 and PD1 on splenic CD4^+^ T cells from young (4–14-week old) wt, DKO, and BXSB-*Yaa* mice. *F* female, *M* male. Data are representative of two independent experiments (*n* = 3–4). **d** Representative FACS plots for BTLA and PD1 gated on CD4^+^CD44^+^ T cells from DKO or BXSB-*Yaa* mice. **e** Representative FACS histograms showing p-4E-BP levels in T_FH_ effector (CD4^+^BTLA^hi^PD1^hi^CD44^+^) and non-T_FH_ effector (CD4^+^BTLA^−^PD1^−^CD44^+^) cells of the indicated genotypes. *Rapa* rapamycin. **f** Western blotting analysis of the levels of Bcl6 in nuclear extracts from naive wt or DKO T cells cultured as in Fig. 3 and treated with vehicle control or the indicated doses of 4EGI-1 for the final 24 h of the 3-day culture. Levels of IRF4 and HDAC1 were evaluated as control. Data are representative of two independent experiments

mTORC1 is a crucial controller of protein synthesis due to its ability to modulate the activity of translational regulators, such as 4E-BP proteins (4E-BP1, 4E-BP2, and 4E-BP3) and S6K1^9, 10^. To assess whether mTORC1 controls the expression of Bcl6 protein in DKO T cells, we utilized rapamycin, a well-known mTORC1 inhibitor. While rapamycin treatment did not affect the low levels of Bcl6 protein in wt T cells, it markedly decreased the high levels of Bcl6 protein in DKO T cells (Fig. 4a and Supplementary Fig. 5a). The inhibitory effects of rapamycin were not due to effects on *Bcl6* transcription (Supplementary Fig. 5b) and did not represent global effects on protein translation since the expression of IRF4 was unaffected by rapamycin treatment (Fig. 4a). Thus the increased expression of Bcl6 protein in DKO T cells is dependent on mTORC1 activity.

The finding that mTORC1 regulated the abundance of Bcl6 protein suggested that mTORC1 activation might be dysregulated in DKO T cells. An examination of the phosphorylation status of 4E-BP proteins, key downstream effectors of mTORC1, demonstrated that, as compared to wt T cells, DKO T cells exhibited higher levels of 4E-BP phosphorylation, which was sensitive to rapamycin inhibition (Fig. 4b, Supplementary Fig. 5c). Higher phosphorylation levels were particularly noticeable in the case of the faster migrating form of p-4E-BP proteins, which may represent phosphorylation of the 4E-BP2 isoform^29^. The phosphorylation levels of S6K1 were also increased in DKO T cells but to a lesser extent (Supplementary Fig. 5d). To further evaluate the activation of mTORC1 in wt and DKO mice, we employed intracellular staining for p-4E-BP proteins. Consistent with the in vitro findings, we observed high levels of 4E-BP phosphorylation in T_FH_ cells derived from DKO mice and in T_FH_ cells from lupus-prone BXSB-*Yaa* mice but not in wt naive T cells (Figs. 4c–e and Supplementary Figs. 5e, f). T_FH_ cells generated upon immunization of wt mice with a T-dependent antigen also exhibited high levels of 4E-BP phosphorylation (Supplementary Fig. 5g). Consistent with previous work showing that T cells from SLE patients exhibit elevated mTORC1 activity including increased 4E-BP phosphorylation^30, 31^, these data indicate that mTORC1 activity is dysregulated in DKO T cells and that high levels of phosphorylation of the mTORC1 target, 4E-BP, characterize the spontaneous accumulation of T_FH_ cells in two different murine lupus models.

4E-BP proteins regulate cap-dependent translation by binding to eIF4E, a key rate-limiting factor in translation initiation, disrupting its interaction with eIF4G, and thereby preventing the formation of the eIF4F complex^32, 33^. Although eIF4E is involved in the translation of all mRNAs, changes in its levels and/or activity can selectively alter the translation of a subset of “eIF4E-sensitive” mRNAs without impacting global protein synthesis^32^. The function of 4E-BP can be mimicked by a small-molecule inhibitor, 4EGI-1, which binds to eIF4E and interferes with its interaction with eIF4G, preferentially inhibiting the translation of “eIF4E-sensitive” mRNAs^33^. To evaluate whether the increased Bcl6 protein expression in DKO T cells depends on eIF4E, T cells from wt and DKO mice were cultured in the presence or absence of 4EGI-1. Expression of the Bcl6 protein in DKO T cells decreased significantly upon exposure to 4EGI-1 in a dose-dependent manner (Fig. 4f, Supplementary Fig. 5h). This effect was not due to a decrease in *Bcl6* mRNA, which, in line with the known ability of Bcl6 to negatively regulate its own expression^7^, increased upon 4EGI-1 treatments (Supplementary Fig. 5i). The expression of IRF4 and HDAC1 was not affected by 4EGI-1 treatments, suggesting that global protein synthesis was not altered by these doses of the inhibitor (Fig. 4f). These data thus support the idea that increased Bcl6 protein levels in DKO T cells are secondary to a deregulated mTOR–4E-BP axis, leading to aberrant Bcl6 protein expression due to eIF4E hyperactivity.

### Def6 inhibits assembly of a raptor-p62–TRAF6 complex

**Fig. 5:**
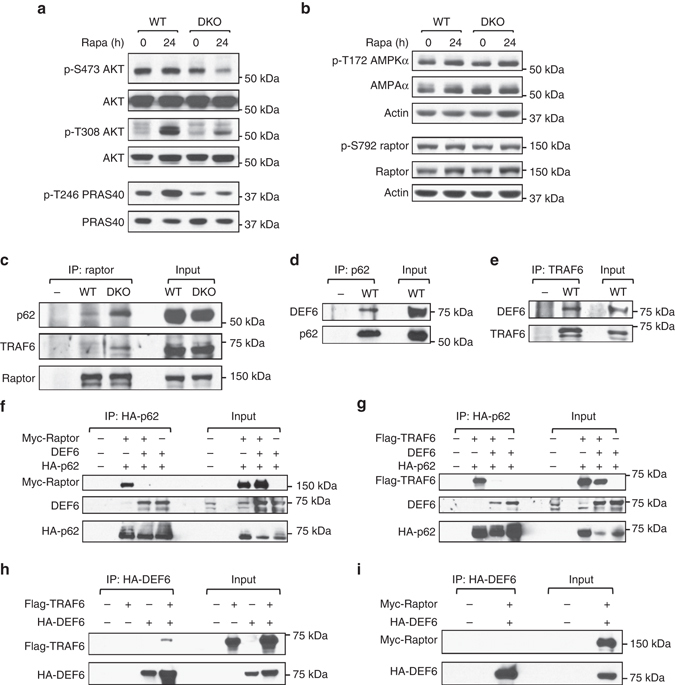
Def6 inhibits assembly of the Raptor–p62–TRAF6 trimolecular complex. **a**, **b** Naive T cells from wt and DKO mice cultured as described in Fig. 3 were treated with vehicle control or rapamycin (20 nM) for the final 24 h of the 3-day culture. The expression levels and phosphorylation status of AKT and PRAS40 **a** and AMPKα and Raptor **b** were assessed by western blotting. **c** Assembly of the raptor–p62–TRAF6 trimolecular complex was evaluated by western blotting after Raptor immunoprecipitation using extracts from wt and DKO T cells. As a control, Raptor antibody immunoprecipitates in the absence of extracts were analyzed along with extracts as input. **d** The interaction between endogenous Def6 and p62 was determined by p62 immunoprecipitation using extracts from wt T cells. **e** The interaction between endogenous Def6 and TRAF6 was determined by TRAF6 immunoprecipitation as in **d**. **f**, **g** 293T cells were cotransfected with expression vectors for Myc-Raptor and HA-p62 **f** or Flag-TRAF6 and HA-p62 **g**, with or without Def6 expression vector. HA-p62 immunoprecipitates were analyzed by western blotting with the indicated antibodies to determine the effect of Def6 on the interaction between Raptor and p62 **f** or p62 and TRAF6 **g**. Data are representative of two independent experiments. **h**, **i** 293T cells were cotransfected with HA-Def6 and either Flag-TRAF6 **h** or Myc-raptor **i**. Cell extracts were immunoprecipitated with anti-HA antibody. The interaction between Def6 and either TRAF6 **h** or raptor **i** was determined by western blotting with the indicated antibodies

mTORC1 can be activated by growth factors, energy status, and availability of amino acids. To dissect the mechanisms leading to mTORC1 activation in DKO T cells, we first focused on the PI3K–AKT axis, the key pathway responsible for growth factor-mediated activation of mTORC1 in T cells^9, 10^. DKO T cells exhibited lower levels of AKT phosphorylation on Ser473 than wt T cells (Fig. 5a). Phosphorylation of AKT on Thr308 was absent in both wt and DKO T cells although it was upregulated after rapamycin treatment, consistent with the known interference of rapamycin with mTORC1-dependent feedback inhibitory mechanisms (Fig. 5a). Consistent with AKT activation levels, the phosphorylation of the AKT target site in PRAS40 (Thr246) was lower in DKO T cells than in wt T cells (Fig. 5a). Thus the PI3K–AKT axis is not responsible for the deregulated mTORC1 activation in DKO T cells.

To determine the contribution of energy-dependent pathways, we assessed the activation state of AMPK, an energy sensor that monitors the intracellular AMP/ATP ratio. AMPK is activated by phosphorylation at Thr172 by liver kinase B1 (LKB1) and, in turn, can phosphorylate raptor at Ser792, inducing its dissociation from mTORC1 and leading to mTORC1 inactivation^10^. The phosphorylation levels of both AMPK Thr172 and raptor Ser792 were similar in T cells from wt and DKO mice, suggesting that the regulation of mTORC1 by energy-dependent pathways is not altered in DKO T cells (Fig. 5b).

Interaction of raptor with p62 and TRAF6 has been implicated in the regulation of nutrient-sensitive pathways that lead to the recruitment of mTORC1 to the lysosomes^12, 13^. To examine this pathway, we first assessed whether assembly of the raptor–p62–TRAF6 complex was appropriately regulated in DKO T cells. Only a minimal interaction of raptor with either p62 or TRAF6 was observed in wt T cells (Fig. 5c). In contrast, raptor strongly interacted with both p62 and TRAF6 in DKO T cells (Fig. 5c). Thus formation of the raptor–p62–TRAF6 complex, a critical step in mTORC1 activation, is deregulated in T cells from DKO mice.

Since T cells lacking Def6 alone but not those lacking SWAP-70 alone exhibited deregulated Bcl6 protein expression (Supplementary Fig. 4c), we next explored the possibility that Def6 controls the assembly of this trimolecular complex. Def6 coimmunoprecipitated with p62 and TRAF6 in wt T cells, indicating that endogenous Def6 interacts with components of this complex under physiological settings (Figs. 5d, e). To further dissect the role of Def6 in the formation of the raptor–p62–TRAF6 complex, we cotransfected HA-tagged p62 with Myc-tagged raptor in 293T cells in the presence/absence of Def6 (Fig. 5f). As expected, in the absence of Def6, raptor coimmunoprecipitated with p62. Consistent with the results observed with the endogenous proteins, Def6 and p62 strongly interacted (Fig. 5f). Strikingly, the presence of Def6 completely abrogated the interaction between raptor and p62 (Fig. 5f). When HA-tagged p62 was cotransfected with Flag-tagged TRAF6, interaction between p62 and TRAF6 was also significantly diminished by the presence of Def6 (Fig. 5g). Cotransfection of HA-tagged Def6 with either Flag-tagged TRAF6 or Myc-tagged raptor moreover demonstrated that Def6 directly interacts with TRAF6 but not with raptor (Figs. 5h, i). A mutational analysis mapped the association of p62 with Def6 to amino acids 225–266, a region of p62 that contains the TRAF6-binding sequence (Supplementary Figs. 6a, b). By directly interacting with p62 and TRAF6, Def6 can thus prevent the assembly of a raptor–p62–TRAF6 complex and inhibit the activation of mTORC1.

### mTOR regulates a subset of proteins in DKO T cells

**Fig. 6:**
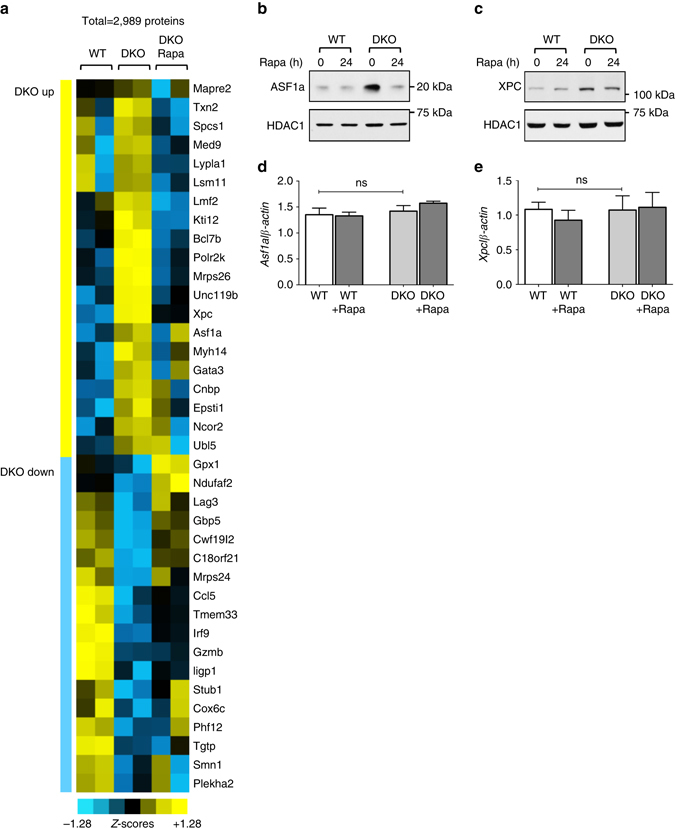
Proteomics-based identification of mTORC1 targets in DKO T cells. **a** Nuclear extracts from naive wt T and DKO T cells were cultured as described in Fig. 3 and treated with vehicle control or rapamycin (20 nM) for the final 24 h of the 3-day culture were subjected to LC-MS/MS. Heatmap of the 20 mTORC1-regulated proteins whose levels were upregulated in DKO T cells as compared to wt and were downregulated upon rapamycin treatment, and the 18 mTORC1-regulated proteins that were downregulated in DKO T cells and whose downregulation was rapamycin sensitive. *Yellow bars* indicate upregulated proteins and *blue bars* indicate downregulated proteins. Color codes for the *Z*-scored LFQ values are shown. Proteins were selected based on the criteria that one comparison needed to be statistically significant by >4-fold difference and that both comparisons needed to be at least >1.7-fold different. **b**, **c** Representative western blottings from at least two independent experiments of mTOR-regulated proteins, ASF1a and XPC. **d**, **e** Quantitative RT–PCR analysis of the expression of *Asf1a* and *Xpc* mRNA in sorted naive T cells cultured as in Fig. 3 and treated with control or rapamycin (20 nM) for the final 24 h of the 3-day culture. The data were normalized relative to *β-Actin* mRNA expression and are combined from three independent experiments. *Error bars* indicate mean±s.d., statistical significance was determined using Wilcoxon matched-pairs test. *ns* not significant

To identify additional proteins that might be aberrantly regulated in DKO T cells in an mTOR-dependent manner, we employed a global proteomic approach. Given that our metabolic labeling experiments had revealed that enhanced [^35^S]methionine incorporation by DKO T cells could be primarily observed in nuclear extracts, we focused on this cellular compartment. Nuclear extracts from biological replicates were obtained from wt T cells and DKO T cells as well as DKO T cells treated with rapamycin. The different samples were subjected to in-solution trypsin digestion followed by liquid chromatography-tandem mass spectrometry (LC-MS/MS) based proteomics analysis. Using label free quantitation (LFQ) we quantitated 2989 proteins. Statistical analysis led to the identification of 20 proteins whose expression was upregulated in DKO T cells in an mTOR-dependent manner (Fig. 6a and Supplementary Data 1). Eighteen proteins were expressed at lower levels in DKO T cells than in wt T cells and their downregulation was rapamycin-sensitive (Fig. 6a and Supplementary Data 1). Performing the classification without imputation revealed an additional seven proteins that were upregulated in DKO T cells in an mTOR-dependent manner and an additional six proteins, whose abundance was lower in DKO T cells than in wt T cells and whose downregulation was rapamycin-sensitive (Supplementary Data 1).

Several of the identified proteins were previously shown to be translationally regulated mTOR targets, such as MYH14 and LMF2 (Fig. 6a and Supplementary Data 1)^34, 35^. The expression of the Bcl6 interaction partner SMRT (also known as NCOR2)^36^ was also increased in DKO T cells in an mTOR-dependent manner (Fig. 6a and Supplementary Data 1). Furthermore, a number of proteins involved in the DNA damage response like ASF1a and XPC^37, 38^ were identified in this analysis (Fig. 6a and Supplementary Data 1). The expression pattern of some of the targets identified in the MS experiment, ASF1a and XPC, was further corroborated by western blotting (Figs. 6b, c). Quantitative real-time PCR for *Asf1a* and *Xpc* confirmed that the differences in expression levels observed in the different samples were not due to transcriptional effects (Figs. 6d, e). Aberrant activation of mTORC1 in DKO T cells thus targets the expression of a subset of proteins in addition to Bcl6.

### mTORC1 regulates T_FH_ cell expansion in lupus-prone mice

**Fig. 7:**
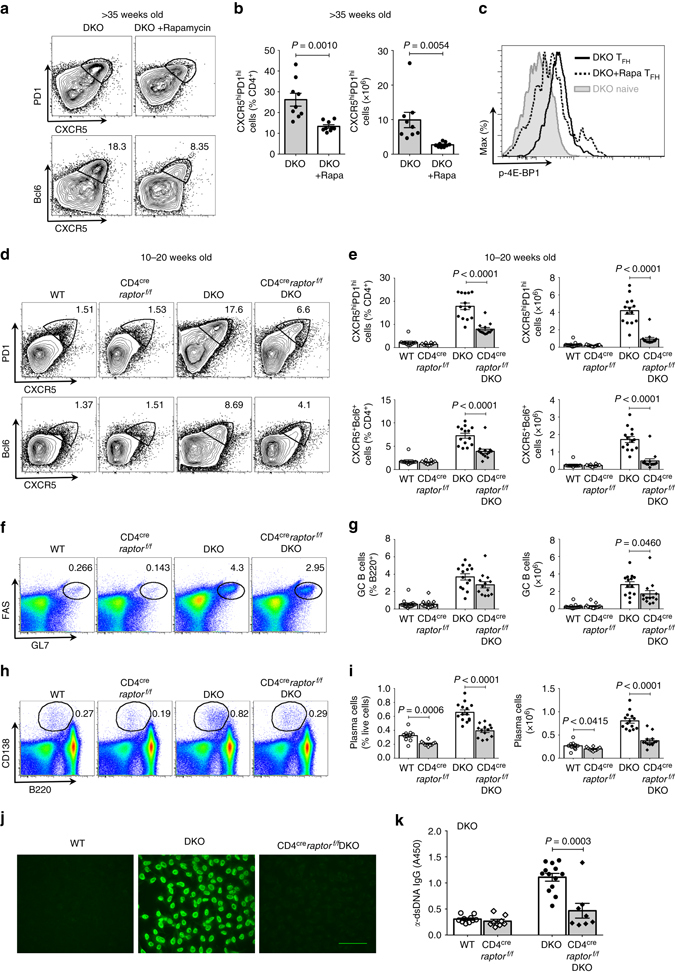
mTORC1 regulates the aberrant T_FH_ cell expansion observed in lupus-prone DKO mice. **a**–**c** T_FH_ cells were analyzed by FACS from aging (>35-week old) DKO mice treated with vehicle control or rapamycin (3 mg/kg) daily for 10 days. **a** Representative FACS plots for CXCR5 and PD1 (*upper* panel) or CXCR5 and Bcl6 (*lower* panel) gated on CD4^+^ T cells. **b** Quantification of T_FH_ cells in DKO mice treated with or without rapamycin (combined data from two independent experiments. *n* = 9 for control mice and *n* = 8 for rapamycin-treated mice). Each *dot* represents an individual mouse. **c** FACS histograms of p-4E-BP levels on CD4^+^CD44^+^BTLA^hi^PD1^hi^ T_FH_ cells or CD4^+^CD44^−^PD1^−^ naive T cells. **d**–**i** Flow cytometric analysis of T_FH_ cells, GC B cells and plasma cells in the spleens of 10–20-week-old wt, CD4^cre^ *raptor* ^*f/f*^, DKO, and CD4^cre^ *raptor* ^*f/f*^DKO mice (*n* = 10–14). Wt mice include 5 CD4cre and 5 C57BL/6 mice while DKO mice include 3 *raptor* ^*f/f*^DKO and 11 DKO mice. **d** Representative FACS plots for CXCR5 and PD1 (*upper* panel) or CXCR5 and Bcl6 (*lower* panel) gated on CD4^+^ T cells from wt, CD4^cre^ *raptor* ^*f/f*^, DKO, and CD4^cre^ *raptor* ^*f/f*^DKO mice. **e** Frequencies and numbers of T_FH_ cells (CD4^+^CXCR5^hi^PD1^hi^; *top* panel, CD4^+^CXCR5^+^Bcl6^+^; *bottom* panel) in wt, CD4^cre^ *raptor* ^*f/f*^, DKO, and CD4^cre^ *raptor* ^*f/f*^DKO mice (*n* = 10–14). **f** Representative FACS plots for GL7 and FAS levels on B220^+^ cells. **g** Quantification of GC B cells (GL7^+^FAS^+^B220^+^). **h** Representative FACS plots for B220 and CD138 gated on live cells from the spleens of wt, CD4^cre^ *raptor* ^*f/f*^, DKO, and CD4^cre^ *raptor* ^*f/f*^DKO mice. **i** Quantifications of plasma cells in wt, CD4^cre^ *raptor* ^*f/f*^, DKO, and CD4^cre^ *raptor* ^*f/f*^DKO mice (*n* = 10–14). Combined data from four independent experiments. **j** ANAs in the sera of aged (>29 weeks) wt, DKO, and CD4^cre^ *raptor* ^*f/f*^DKO mice (*n* = 5, 8, 8) were analyzed by indirect immunofluorescence. Representative images from two independent experiments are shown. *Scale bar*, 100 μm. **k** Anti dsDNA-specific IgG levels in the sera of aged (>29 weeks) wt, CD4^cre^ *raptor* ^*f/f*^, DKO, and CD4^cre^ *raptor* ^*f/f*^DKO mice (*n* = 10, 8, 13, 8) were assessed by ELISA. Each *dot* represents an individual mouse. *Error bars* indicate mean ± s.d., *P*-value by unpaired *t*-test

To start assessing whether abnormalities in mTORC1 activation in DKO T cells contributed to the aberrant expansion of T_FH_ cells in DKO mice, we next evaluated whether inhibition of mTORC1 by rapamycin treatment could decrease the accumulation of T_FH_ cells in these mice. Daily injection of rapamycin intraperitoneally for 10 days into aging DKO female mice resulted in a significant reduction in the frequency and numbers of T_FH_ cells in DKO mice (Figs. 7a, b and Supplementary Fig. 7a). Consistent with this finding, phosphorylation of 4E-BP in DKO T_FH_ cells was significantly reduced by rapamycin treatment (Fig. 7c). The changes in the T_FH_ cell compartment were accompanied by a profound decrease in GC B cells although total B-cell numbers were unaffected (Supplementary Figs. 7b–d). These studies thus support the idea that mTORC1 activation plays a critical role in the aberrant humoral responses of DKO mice.

Given that rapamycin could affect the activation of mTORC1 in multiple cellular compartments, we then proceeded to directly evaluate the impact of T cell–mTORC1 in DKO mice by selectively deleting raptor in the DKO T-cell compartment. As an initial step, we generated CD4^cre^ *raptor* ^*f/f*^ mice, lacking raptor in CD4^+^ T cells^39^ and immunized them with a T-dependent antigen (NP-KLH). T_FH_ cells were decreased in CD4^cre^ *raptor* ^*f/f*^ mice (Supplementary Figs. 7e, f). We then crossed CD4^cre^ *raptor* ^*f/f*^ mice to DKO mice to generate CD4^cre^ *raptor* ^*f/f*^DKO mice. In line with the notion that Bcl6 is necessary to drive T_FH_ cell differentiation and that mTORC1 regulates Bcl6 in DKO T cells, CD4^cre^ *raptor* ^*f/f*^DKO mice exhibited fewer CXCR5^hi^PD1^hi^ CD4^+^ T cells and CXCR5^hi^Bcl6^hi^ CD4^+^ T cells, indicating that expansion of this compartment in DKO mice is mTORC1-dependent (Figs. 7d, e, Supplementary Figs. 7g, h). T cells producing both IL-21 and IFN-γ were also significantly decreased in CD4^cre^ *raptor* ^*f/f*^DKO mice (Supplementary Fig. 7i). Interestingly, an analysis of the B-cell compartment in CD4^cre^ *raptor* ^*f/f*^DKO mice revealed that plasma cells in these mice were reduced to a greater extent than GC B cells (Figs. 7f–i and Supplementary Figs 7j, k). Notably, autoantibody production was significantly diminished in CD4^cre^ *raptor* ^*f/f*^DKO mice (Figs. 7j, k), confirming a crucial role for T-cell mTORC1 in the accumulation of T_FH_ cells and the systemic autoimmune responses that characterize the DKO mice.

## Discussion

The acquisition of a T_FH_ cell phenotype is critically dependent on Bcl6^3–5^ and aberrancies in T_FH_ cell homeostasis have been linked to autoimmune disorders, including SLE^1, 2^. While previous studies have elegantly outlined the transcriptional control of *Bcl6* expression^6^, here we demonstrate that the total levels of Bcl6 in T cells can also be regulated by controlling the de novo synthesis of the Bcl6 protein in an mTORC1-dependent manner. We furthermore show that mTORC1 activation in this autoimmune setting proceeds via an alternative pathway, which involves the assembly of a raptor–p62–TRAF6 complex, and controls the abundance of a selected group of proteins. Thus, in addition to effects on the transcriptional landscape and the metabolic programs of T cells, the ability of mTORC1 to regulate the expression of lineage-defining transcription factors by controlling protein synthesis can also be an important contributor to T-cell dysfunction and the development of autoimmunity.

The ability of 4EGI-1, an inhibitor of the eIF4E-eIF4G interaction, to downregulate the expression of the Bcl6 protein in DKO T cells supports the idea that this Ragulator-independent docking system may be particularly important for the regulation of the mTORC1–4E-BP–eIF4E axis. Notably, eIF4E has been shown to enhance the translation of a specific group of transcripts during cellular stress and knockdown of eIF4E selectively affects these transcripts without global decreases in protein levels^40^. Transcripts that are particularly dependent on eIF4E levels are believed to contain highly structured 5′ untranslated regions that require unwinding by eIF4A and/or encode proteins, which, like Bcl6, have a short half-life. In line with our data, eIF4E was recently shown to control the abundance of the Bcl6 protein in diffuse large B-cell lymphomas^41^. Thus employment of this alternative pathway to regulate mTORC1 activity could permit T_FH_ cells to maintain optimal translation of selected key targets, like Bcl6, Ncor2, and DNA damage response proteins, in the GC light zones where hypoxic conditions have recently been shown to occur^42^. In line with this notion, increased levels of 4E-BP phosphorylation could be detected in T_FH_ cells generated upon immunization of wt mice with a T-dependent antigen. Inappropriate control of this mTORC1–4E-BP–eIF4E axis, in turn, may enable autoimmune T_FH_ cells to bypass an important checkpoint and endow them with enhanced competitive fitness.

Strong T-cell receptor (TCR) engagement is a key controller of the acquisition of a T_FH_ cell phenotype^43^ and the requirement for distinct APC–T-cell interactions can be altered by the presence of an abundant source of antigen^44^. Def6 contains multiple phosphorylation sites, which can be phosphorylated by Lck and ITK, and undergoes profound conformational changes upon TCR engagement^27, 28^. In particular, ITK-mediated phosphorylation of Def6 promotes its aggregation and the formation of Def6 cytoplasmic granules that co-localize to P-bodies^45^. The involvement of Def6 in controlling assembly of the raptor–p62–TRAF6 complex could thus provide a direct mechanistic link by which TCR engagement could regulate the mTORC1–4E-BP–eIF4E axis. By post-translationally modifying Def6, T cells could selectively change the inhibitory effects of Def6 on the p62–TRAF6 interaction. Indeed, Def6 and p62 exhibited a similar localization in T_FH_ cells from immunized wt mice (Supplementary Fig. 8a). Intriguingly the expression of Slc7a5 and other amino-acid transporters is also controlled by TCR engagement and the lack of Scl7a5 results in defective high-affinity T-dependent humoral responses^46^. Thus TCR engagement may control this axis via its dual ability to regulate Def6 activity and amino-acid availability.

The capacity of Def6 to control protein synthesis in addition to cytoskeletal reorganization and gene expression^27, 28^ supports the idea that employment of multifunctional signaling hubs like Def6 may enable T cells to precisely coordinate these critical processes in a rapid manner when faced with quickly changing environmental conditions such as those encountered in the GCs. Importantly, the involvement of Def6 in the integration of these diverse processes may be a crucial feature underlying the ability of *Def6* variants to be associated with the pathogenesis of human SLE. In this regard, the availability of mouse models where the lack of Def6 leads to the development of a disease that recapitulates key features of human SLE, like the aberrant expansion of T_FH_ cells, can provide an invaluable platform for an in-depth understanding of the mechanisms by which human SLE variants could impact disease pathogenesis.

Recent studies have uncovered a surprising level of complexity in the reliance of T_FH_ cells on the mTOR pathway with differential requirements for the mTORC1 complex depending on the stimulus triggering their formation and their localization^14–16^. We now show that, despite the well-known ability of autoimmune T cells to deregulate multiple signaling pathways^47^, a feature that can potentially lessen their dependency on any one pathway, the inappropriate accumulation of T_FH_ cells in the chronic inflammatory environment characteristic of autoimmune settings requires T cell–mTORC1. It is likely that this dependency results from both a direct role of mTORC1 in maintaining high levels of Bcl6 protein expression and from the known effects of mTORC1 on T-cell metabolism^48–50^. Importantly, the T_FH_ cells that expand in DKO mice remain amenable to therapeutic interventions aimed at inhibiting mTORC1 since administration of rapamycin to aging DKO mice results in a decreased T_FH_ compartment and a marked and rapid amelioration of their exuberant humoral responses. Given that rapamycin is being investigated as a treatment for SLE^17^, these findings could have important therapeutic implications and help define the patient subsets that might be most responsive to this treatment.

Our data thus indicate that aberrancies in mechanisms controlling de novo protein synthesis represent a novel mechanism leading to T-cell dysfunction in autoimmunity. The ability of post-transcriptional mechanisms to selectively modify the expression of key regulators of T_H_ cell fate decisions furthermore suggest that, in order to fully understand the functional phenotype of autoimmune T cells, transcriptomic and epigenomic evaluations will need to be complemented by proteomic analyses. Together with previous work demonstrating that abnormalities in mRNA stability can also lead to deregulation of the T_FH_ cell compartment^2, 51^, post-transcriptional mechanisms may thus emerge as major contributors to ensure T_FH_ cell homeostasis.

## Methods

### Mice

C57BL/6, B6. SJL (CD45.1), *Rag1* ^*−/−*^, CD4^cre^, *raptor* ^*f/f*^ ^52^, and BXSB-*Yaa* mice were obtained from Jackson Laboratory. *Def6*-deficient (Def6^*tr/tr*^) mice were generated by Lexicon Pharmaceuticals, Inc. using a gene trapping strategy as previously described^21^ and then backcrossed onto C57BL/6 background for >10 generations. *Swap70*-deficient mice (*Swap70* ^*−/−*^) were generated by R. Jessberger as previously described^21^. *Def6* ^*tr/tr*^ *Swap70* ^*−/−*^ (DKO) mice were generated by crossing *Def6* ^*tr/tr*^ mice with *Swap70* ^*−/−*^ mice that had been backcrossed onto C57BL/6 background for >10 generations. All the mice used in the experiments were females unless stated otherwise and housed in a specific pathogen-free animal facility in the Hospital for Special Surgery. Experiments were performed according to protocols approved by the Institutional Animal Care and Use Committee of the Hospital for Special Surgery.

### Flow cytometry

For surface staining, cells were incubated with antibodies at 4 °C in flow cytometry buffer. Antibodies to CD4 (RM4^−^5; 400×), ICOS (C398.4A; 200×), B220 (RA3-6B2; 400×), and CD44 (IM7; 1,000×) were obtained from Biolegend. Antibodies to Bcl6 (K112-91; 50×), CD138 (281-2; 400×), GL-7 (800×), and Fas (Jo2; 200×) were obtained from BD. Antibodies to CD45.2 (104; 200×), and PD1 (J43; 200×) were obtained from eBioscience. For staining of CXCR5 (2G8; 200×; BD), cells were incubated in dark at room temperature for 25 min. For intracellular staining, cells were fixed after the surface staining at 4 °C with Cytofix/Cytoperm buffer (BD) and washed twice with Perm/Wash buffer (BD). For Foxp3 staining, cells were fixed and permeabilized with a Mouse Regulatory T-cell staining Kit (FJK-16s; eBioscience) according to the manufacturer’s instructions. For intracellular cytokine staining, splenocytes were stimulated with 50 μg/ml phorbol myristate acetate and 1 μM Ionomycin (EMD) for 4 h. The cells were incubated with 1 μM Monensin (eBioscience) for the final 3 h of stimulation. After stimulation, cells were subjected to intracellular staining for cytokines using anti-mouse IL-17 (eBio17B7; 600×), IFN-γ (XMG1.2; 1,000×; eBioscience), and recombinant mouse IL-21R Fc Chimera (R&D Systems; 1 μg/ml) followed by phycoerythrin-labeled affinity-purified F(ab’)_2_ fragment of goat anti-human Fcγ (Jackson ImmunoResearch Laboratories; 250×). Flow cytometric data were analyzed with the FlowJo software (TreeStar).

For phospho-flow cytometry, splenocytes were fixed with 2% paraformaldehyde at 37 °C. After fixation, cells were chilled on ice followed by Fcγ blockade (MSKCC; 1,000×). After surface staining with antibodies to CD4 (RM4-5), CD44 (IM7), PD1 (J43), and BTLA-4 (8F4; 400×), cells were permeabilized with 90% ice-cold methanol. The cells were blocked with 10% fetal calf serum/phosphate-buffered saline (PBS) for 10 min at room temperature followed by intracellular staining for p-4E-BP (Cell Signaling Technology; 50×) for 45 min at room temperature.

### Immunofluorescence microscopy

Spleens were embedded in OCT (Tissue-tek, Sakura) and frozen in 2-methylbutane surrounded by dry ice. Frozen blocks were cut into sections 6 μm in thickness with cryotome and dried >2 h at room temperature. Sections were fixed in cold acetone for 15 min and kept at −80 °C. Nonspecific binding was blocked for 15 min with 3% bovine serum albumin (BSA) in PBS, and then sections were stained with PNA-FITC (Vector Laboratory; 200×), anti-PD1 (RMP1-14; 50×; Biolegend), and anti-CD3-APC (2C11; 50×; eBioscience) for 25 min at room temperature in dark. Cy3-labeled donkey anti-rat immunoglobulin G (IgG) (H + L) (200×; Jackson ImmunoResearch, 712-165^-^153) was used to detect the PD1 expression. Slides were mounted using anti-fade reagent (Invitrogen Life Technologies) containing DAPI (4,6-diamidino-2-phenylindole; Invitrogen) before microscopic image acquisition. Images were acquired with a Zeiss Axioplan2 imaging microscope and Adobe Photoshop was used for analysis.

### Bone marrow chimeras

To generate mixed bone marrow chimeras, 8×10^6^ T/B-cell-depleted bone marrow cells were injected into *Rag1* ^*−/−*^ mice, which were lethally irradiated (875 rads). CD45.1^+^ wild-type bone marrow cells were mixed with an equal number of either CD45.2^+^ wt or DKO bone marrow cells and injected into *Rag1* ^*−/−*^ recipients via retro-orbital injection. Recipient mice were analyzed 8–10 weeks after the reconstitution.

### Cell sorting and T-cell stimulation

Single-cell suspensions from the pooled spleens and lymph nodes were enriched for CD4^+^ T cells with CD4 microbeads (Miltenyi Biotech). Naive (CD4^+^CD25^−^CD44^−^ CD62L^+^) T cells were purified from enriched CD4^+^ T cells on a MoFlo cytometer (DakoCytomation). Naive T cells were stimulated with plate-bound anti-CD3 (145-2C11; BioXcell; 10 μg/ml) and soluble anti-CD28 (37.5; BioXcell; 10 μg/ml) at a density of 1×10^6^ cells/ml in complete RPMI medium supplemented with 10% fetal calf serum, 2 mM glutamine, 100 IU/ml penicillin, 0.1 mg/ml streptomycin, 10 mM Hepes, 1× nonessential amino acids (Cellgro), and 50 μM β-mercaptoethanol. Rapamycin was purchased from Cell Signaling Technology and 4EGI-1 was purchased from Selleckchem.

### DNA constructs

Expression plasmids for untagged and HA-tagged Def6 were generated as described previously^21^. Expression constructs for Myc-tagged raptor (Addgene plasmid 1859^53^) and HA-tagged p62 (Addgene plasmid 28027^54^) were purchased from Addgene. Expression plasmids for Flag-tagged TRAF6 and Flag-tagged full-length p62 and its various deletion mutants were constructed in p3XFLAG-CMV-10 expression vector (Sigma) and transfected into 293T (CRL-3216; ATCC) cells.

### Western blotting and immunoprecipitation

Nuclear and cytoplasmic extracts from naive T cells cultured for 3 days were prepared with NE-PER Nuclear and Cytoplasmic Extraction Reagents (Pierce), as previously described^21^. The purity of the nuclear and cytoplasmic fractions was verified by probing with antibodies against Lamin B1 (D4Q4Z; 1,000×; Cell Signaling Technology). Whole-cell extracts were immunoprecipitated with an anti-Raptor (24C12; 100×; Cell Signaling Technology), anti-Def6 (Rabbit polyclonal; 100×^21^), anti-p62 (H-290; 50×; Santa Cruz), anti-TRAF6 (H274; 50×; Santa Cruz), or anti-HA (3F10; 50×; Roche Applied Science) antibodies. Antibodies to p-STAT3 (Y705; 1,000×), p-4E-BP (T37/46; 1,000×), 4E-BP (1,000×), p-S6K1 (S371; 1,000×), S6K1 (1,000×), p-AKT (S473; 1,000×), AKT (1,000×), p-AKT (T308; 1,000×), p-PRAS40 (T246; 1,000×), PRAS-40 (1,000×), p-AMPK (T172; 1,000×), AMPK (1,000×), p-Raptor (S792; 1,000×) and p62 (5114; 1,000×) were obtained from Cell Signaling Technology. Antibodies to IRF4 (M-17; 1,000×), TRAF6 (H274; 500×), and c-Myc (9E10; 500×) were obtained from Santa Cruz. Anti-Bcl6 antibody was obtained from BD (K112-91; 1,000×). Anti-Flag monoclonal antibody M2 (horseradish peroxidase (HRP)) was obtained from Sigma (1,000×).

### [^35^S]-metabolic labeling

Naive T cells cultured for 3 days were starved for 30 min in methionine and cysteine-free RPMI medium (Cellgro). The cells were labeled with [^35^S] methionine/[^35^S] cysteine Labeling Mix (Express Protein Labeling Mix, Perkin Elmer) for 1 h at a final concentration of 50 µCi/ml. After the labeling, cells were washed twice with cold PBS and nuclear extract were prepared with NE-PER Nuclear and Cytoplasmic Extraction Reagents (Pierce). Immunoprecipitation of Bcl6 was performed using a Bcl6 antibody (4242, Cell Signaling Technology). Bcl6 immunoprecipitates were separated by electrophoresis on 8% sodium dodecyl sulfate-polyacrylamide gel electrophoresis. The gel was dried and placed on a film for autoradiography at −80 °C.

### Real-time RT–PCR analysis

Total RNA was extracted from naive cells and cultured for 3 days in vitro with the RNeasy Mini Kit (Qiagen), and cDNA were synthesized with the iScript cDNA Synthesis Kit (BIO-RAD). The gene expression was analyzed by real-time PCR using a SYBR Green PCR Master Mix Kit (Applied Biosystems). The primer pair 1 for *Bcl6* was purchased from Qiagen (QuantiTect Primer Assay, QT01057196). The primer pair 2 for real-time RT–PCR analysis of *Bcl6* and *Hprt* were previously described^55^. The primer pair 2 for *Bcl6* was: forward, 5′-AGGCCTCCTTCCGCTACAAG-3, reverse, 5′-CAAATGTTACAGCGATAGGGTTTCT-3′. The primer pairs for *Hprt* were: forward, 5′-AGCCTAAGATGAGCGCAAGT-3′, reverse, 5′-TTACTAGGCAGATGGCCACA-3′ and *β-Actin*: forward, 5′-GACGGCCAGGTCATCACTATTG-3′, reverse, 5′-AGGAAGGCTGGAAAAGAGCC-3′. The expression of *Bcl6* was normalized to the expression of *β-Actin* or *Hprt*. The primer pairs for *XPC*: forward, 5′-TCCAGGGGACCCCACAAAT-3′, reverse, 5′-GCTTTTTGGGTGTTTCTTTGCC-3′; *Asf1a*: forward, 5′-GTGGTGCTGGATAACCCGTC-3; reverse, 5′-GGGACCCACTAAAACAGAGTCTA-3′; and *Tbx21*: forward, 5′-GTCCAAGTTCAACCAGCACC-3′, reverse, 5′-GTTGGTGAGCTTTAGCTTCC-3′.

### In vivo treatment with rapamycin and immunizations

Rapamycin was obtained from LC Laboratories and dissolved as previously described^56^. Briefly, rapamycin was dissolved in dimethyl sulfoxide to 100 mg/ml, which was diluted in 5% PEG-400 (Sigma) and 5% Tween-20 to 1 mg/ml, and stored in −80 °C. Aging (>24-week old) DKO mice were injected with either rapamycin (3 mg/kg) or vehicle control intraperitoneally daily for 10 days before the analysis. For T-dependent immunizations, 6–10-week-old mice were immunized intraperitoneally with 50 μg NP-CGG or NP-KLH in alum.

### Anti-dsDNA ELISA and antinuclear antibody (ANA) detection

For anti-dsDNA ELISA, plates were coated with 100 μg/ml salmon sperm DNA (Invitrogen AM9680) at 4 °C overnight and blocked in 2% BSA in PBS, at room temperature for 2 h. Sera were diluted 1:200 and incubated on coated plates at room temperature for 2 h. Plates were then incubated with HRP-labeled goat anti-mouse IgG Fc antibody for 1 h (eBioscience). OD_450_ was measured on a microplate reader. ANAs were detected on Hep-2 slides (MBL International) at a 1:200 dilution using Alexa Flour 488-conjugated anti-mouse IgG (Jackson ImmunoResearch).

### LC-MS/MS and proteomic data analysis

Samples were denatured in urea and reduced prior to alkylation of cysteines with iodoacetamide. Proteins were digested with LysC (Wako Chemicals) followed by trypsination (Promega) and desalted^57^. Approximately 2 µg of each sample was injected for nano-LC-MS/MS analysis (QExactive, Thermo Scientific). Peptides were separated using a heated 50 cm×75 µm C18 column packed with 1.8 µm beads (EasySprayer, Thermo Scientific) at a flow rate of 200 nl/min, with a gradient increasing from 1% B to 45% B over 166 min (buffer A 0.1% formic acid, buffer B 0.1% formic acid in acetonitrile). LFQ: LC-MS/MS data from two biological replicates of two samples were analyzed using the MaxQuant (version 1.5.0.30) and Perseus software (version 1.5.0.9)^58^, searching against a Uniprot mouse database (July 2014). Oxidation of methionine and protein N-terminal acetylation were allowed as variable modifications, and cysteine carbamidomethyl were set as a fixed modification. Two missed cleavages were allowed for specificity: Trypsin/P. The “match between runs” option was enabled. False-discovery rates at the protein and peptide level were set to 1%. Protein abundancies are expressed as LFQ values. Only proteins quantified in at least two out of two replicates in at least one group were retained, and missing values were imputed. A multiple sample analysis of variance test was performed and corrected for multiple hypotheses testing a threshold of *P*-value better than 0.05. *P*-value-based *t-*tests for DKO and DKO with rapamycin against wt was performed.

### Statistics

The comparisons between two different groups were done with unpaired two-tailed Student’s *t*-tests. All *P*-values <0.05 were considered significant. Statistical analysis was performed with Graphpad Prism 6. Statistical analysis of the quantitative RT–PCR data of the combined experiments was performed using Wilcoxon matched-pairs test. The quantification of protein intensity in western blotting was done with ImageJ (1.50i).

### Data availability

All data supporting the findings of this study are included in the article, its Supplementary Information Files, or from the corresponding author upon request.

## Electronic supplementary material

Supplementary information

Supplementary dataset 1
