# Supplementary material for: The mTORC1-4E-BP-eIF4E axis controls de novo Bcl6 protein synthesis in T cells and systemic autoimmunity
Source: Nat Commun. 2017 Aug 15;8:254. doi: 10.1038/s41467-017-00348-3 (PMC5557982; doi:10.1038/s41467-017-00348-3)
Supplement: Supplementary file 1 — Supplementary information [file 41467_2017_348_MOESM1_ESM.pdf]

Title of file for HTML: Supplementary Information
Description: Supplementary Figures

Title of file for HTML: Supplementary Data 1
Description: Mass spectrometry data

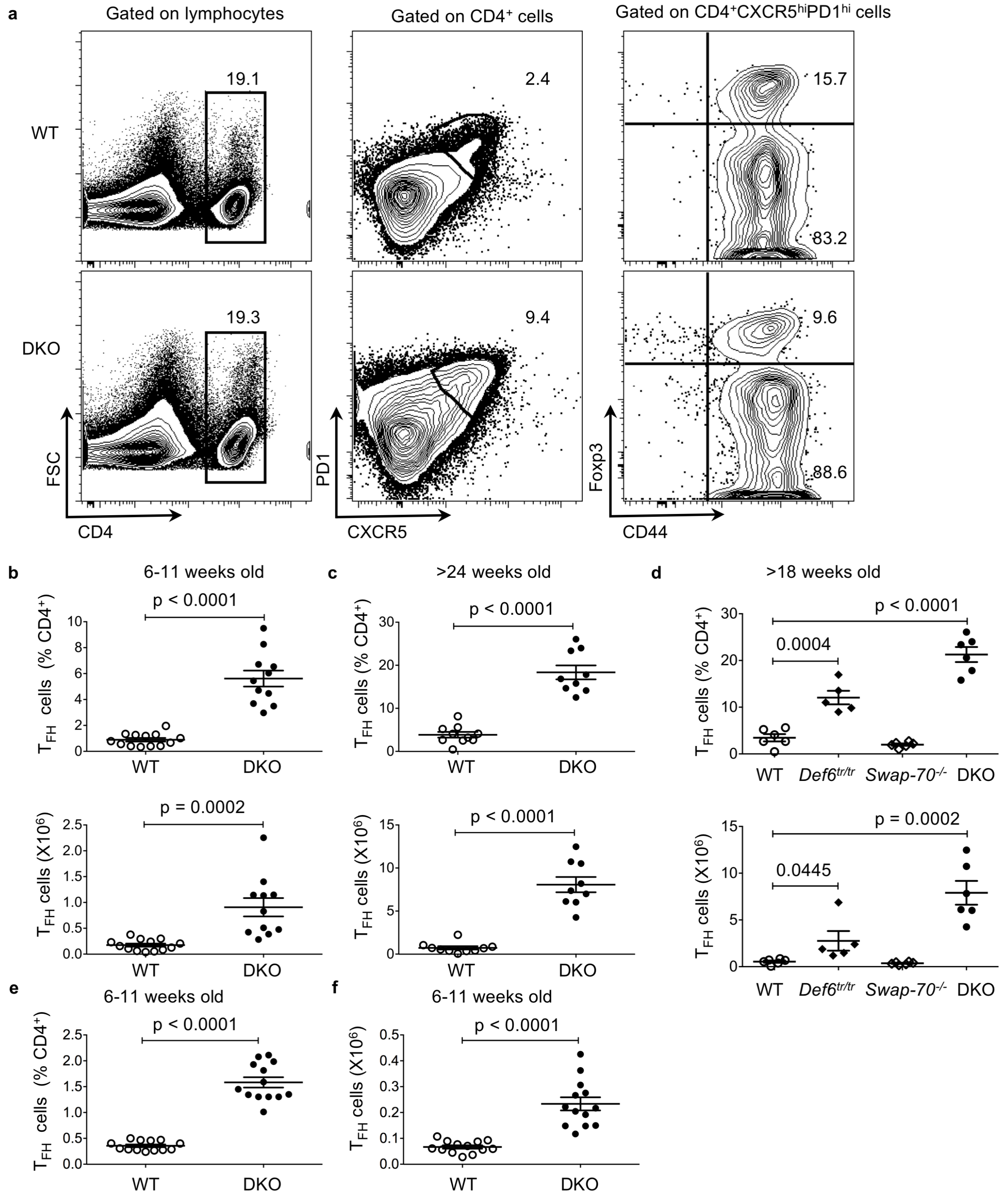

Supplementary Figure 1 | Alternative gating strategy for the identification of T_{FH} cells. (**a**) Representative lymphocyte gates for the identification of T_{FH} cells. T_{FH} cells were analyzed by FACS in the spleens of wild type (upper panel) and DKO (*Def6$^{tr/tr}$Swap-70$^{-/-}$*) (lower panel) mice. After gating on $CD4^+$ T helper cells (left panel), $CD4^+PD1^{hi}CXCR5^{hi}$ cells (middle panel) were defined as T_{FH} cells ($CD4^+CXCR5^{hi}PD1^{hi}CD44^+$). All the T_{FH} cells in this study were evaluated by using this gating strategy. (**b**-**e**) Quantification of the frequencies and numbers of T_{FH} cells ($CD4^+CXCR5^{hi}PD1^{hi}CD44^+Foxp3^-$) after excluding follicular regulatory T cells ($CD4^+CXCR5^{hi}PD1^{hi}CD44^+Foxp3^+$) based on $Foxp3^+$ staining (right upper panel). (**e**,**f**)($CD4^+CXCR5^{hi}PD1^{hi}CD44^+Foxp3^-$) in young (**b**,**e**,**f**) and aging (**c**,**d**) mice of the indicated genotypes.

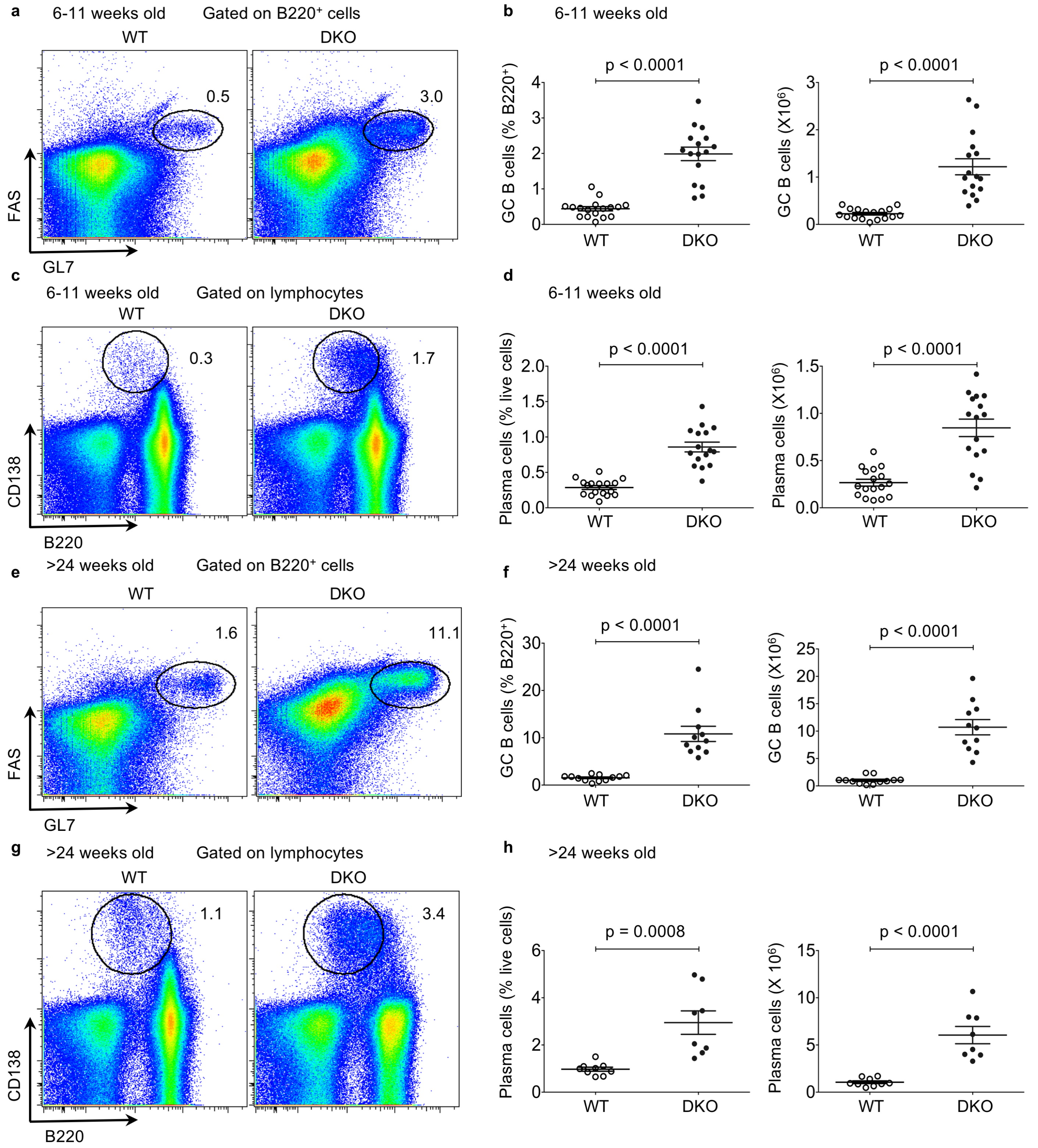

Supplementary Figure 2 | Spontaneous accumulation of GC B cells and plasma cells in DKO mice. GC B cells were analyzed by FACS in the spleens from young (6-11-week-old) (**a**-**d**) and aging (>24 week-old) wt and DKO (*Def6*$^{tr/tr}$*Swap-70*$^{-/-}$) mice (**e**-**h**). WT (open circle), DKO (closed circle). (**a**) Representative FACS plots for GL7 and FAS gated on B220^{+} cells from young mice. (**b**) Quantification of GC B cells in young mice (n=16-17). (**c**) Representative FACS plots for B220 and CD138 gated on live cells from young mice. (**d**) Quantification of plasma cells in young mice (n=16-17). (**e**) Representative FACS plots for GL7 and FAS gated on B220^{+} cells in aging mice. (**f**) Quantification of GC B cells in aging mice (n=11-12). (**g**) Representative FACS plots for B220 and CD138 gated on live cells from aging mice. (**h**) Quantifications of plasma cells in aging mice (n=8-9). Each dot represents an individual mouse. Error bars indicate mean ± s.d., p value by unpaired *t*-test.

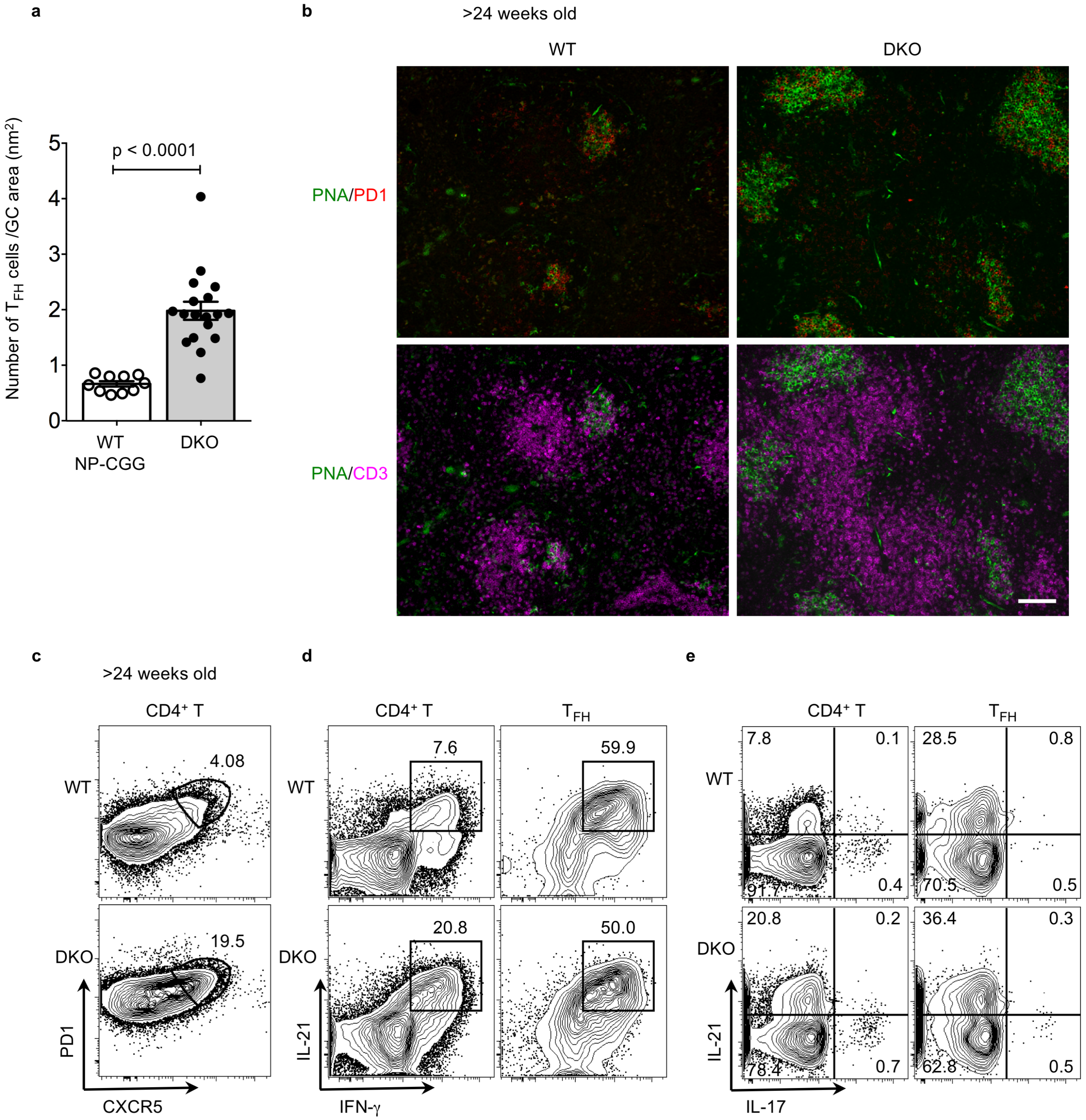

Supplementary Figure 3 | T_{FH} cells are localized in the germinal center and produce IFN-γ and IL-21 in aging DKO mice. (**a**) Quantification of T_{FH} cells in germinal centers from wt mice 8 days post-immunization with NP-CGG and young DKO unimmunized mice. (**b**) Immunofluorescence images of splenic sections from aging (>24 weeks old) wt and DKO (*Def6*$^{tr/tr}$*Swap-70*$^{-/-}$) mice. Representative images from two independent experiments with 2 to 3 mice each. PNA, green; PD1, red; CD3, magenta. Scale bar, 100µm. (**c**-**e**) Representative FACS plots showing intracellular stains of IL-21 and IFN-γ (**d**) or IL-21 and IL-17 (**e**) gated on either CD4^{+} T cells or CXCR5^{hi}PD1^{hi}CD4^{+} T cells (**c**) in the spleens from aging (>24 week-old) mice.

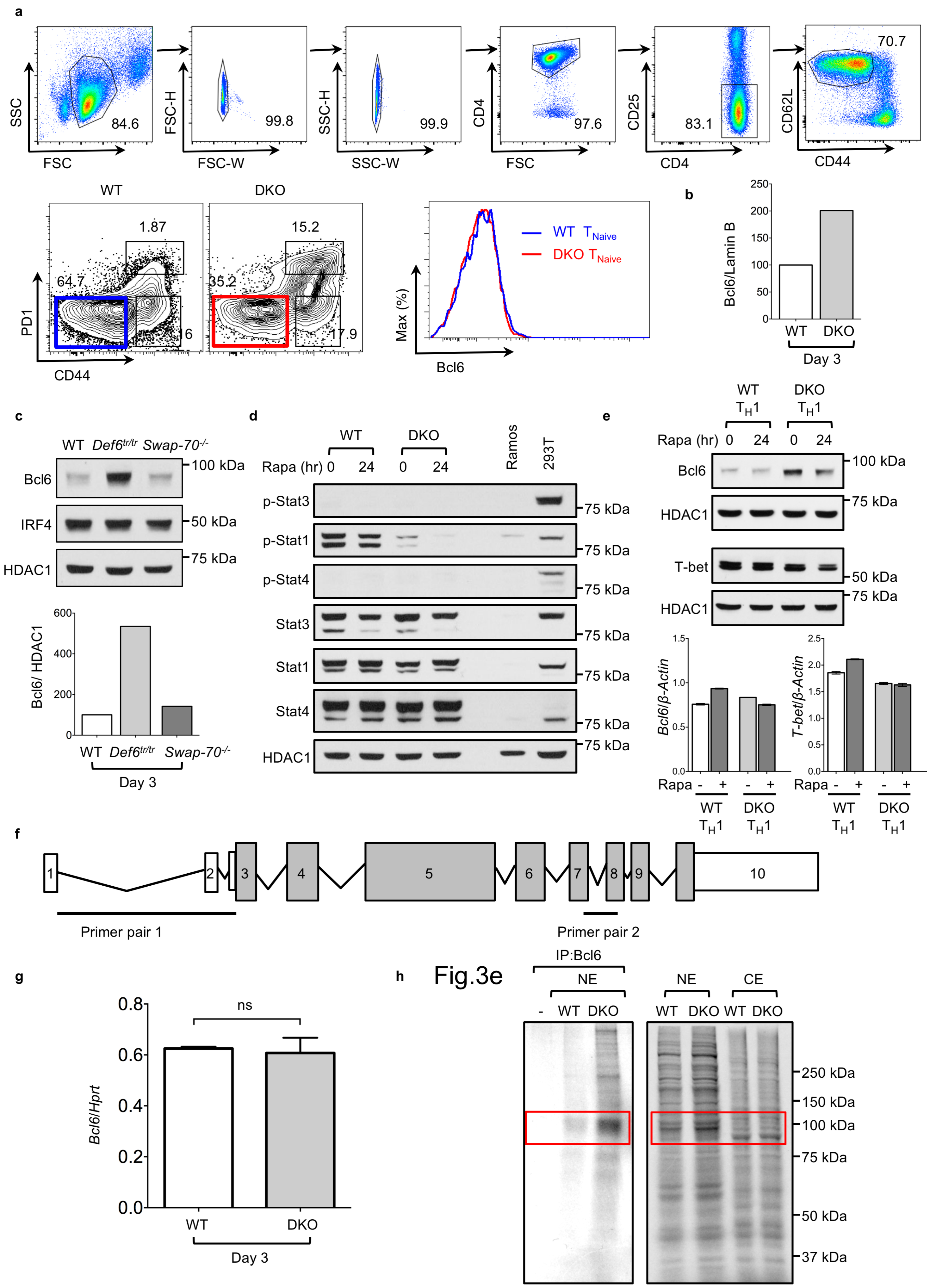
a
SSC
FSC
84.6
FSC-H
FSC-W
99.8
SSC-H
SSC-W
99.9
CD4
FSC
97.6
CD25
CD4
83.1
CD62L
CD44
70.7
WT
DKO
1.87
64.7
15.2
35.2
17.9
PD1
CD44
WT T_Naive
DKO T_Naive
Max (%)
Bcl6
b
Bcl6/Lamin B
WT
DKO
Day 3
c
WT
Def6^tr/tr
Swap-70^-/-
Bcl6
IRF4
HDAC1
100 kDa
50 kDa
75 kDa
Bcl6/ HDAC1
Day 3
d
WT
DKO
Rapa (hr) 0 24 0 24
Ramos
293T
p-Stat3
p-Stat1
p-Stat4
Stat3
Stat1
Stat4
HDAC1
75 kDa
e
WT T_H1
DKO T_H1
Rapa (hr) 0 24 0 24
Bcl6
HDAC1
T-bet
HDAC1
100 kDa
75 kDa
50 kDa
Bcl6/β-Actin
T-bet/β-Actin
Rapa - + - +
f
1
2
3
4
5
6
7
8
9
10
Primer pair 1
Primer pair 2
g
Bcl6/Hprt
ns
WT
DKO
Day 3
h
Fig.3e
IP:Bcl6
NE
CE
- WT DKO
WT DKO WT DKO
250 kDa
150 kDa
100 kDa
75 kDa
50 kDa
37 kDa

Supplementary Figure 4 | *Bcl6* mRNA and protein levels in wt and DKO T cells. (**a**) Gating strategy for the sorting of naïve T cells for *in vitro* culture (top panel) and representative FACS plots for CD44 and PD1 (bottom left panel) or FACS histograms of Bcl6 expression (bottom right panel) gated on naive T cells ($CD4^{+}CD44^{-}PD1^{-}$) from spleens of young mice (6-11-week old) of the indicated genotypes. Data are representative of two independent experiments. (**b**) The abundance of Bcl6 protein in Fig. 3a was quantified and normalized to Lamin B. The levels of Bcl6 in the wt samples were set as 100%. (**c**) Naïve T cells from wt, *Def6*$^{tr/tr}$, and *Swap-70*$^{-/-}$ mice were stimulated with anti-CD3 and anti-CD28 for three days. Nuclear extracts were evaluated by Western blotting with antibodies to Bcl6 and IRF4. HDAC1 was used as a loading control (top panel). Quantification of Bcl6 signal intensity is shown (bottom panel). (**d**) Western blots showing the phosphorylation of Stat1, Stat3, and Stat4 in wt and DKO T cells stimulated with anti-CD3 and anti-CD28 for three days. (**e**) Naïve T cells from wt and DKO mice were stimulated with anti-CD3 and anti-CD28 in the presence of IL-12 and anti-IL-4 (T_H1) for three days (top panel). Nuclear extracts were evaluated by Western blotting with antibodies against Bcl6 and T-bet. HDAC1 was used as a loading control (bottom panel). Quantitative RT-PCR analysis of the expression of *Bcl6* and *T-bet* mRNA. The data were normalized relative to *β-Actin* mRNA expression. Data are representative of two independent experiments. (**f**) Schematic representation showing the organization of *Bcl6* primary transcript. Exons are shown as boxes numbered 1-10, whereas introns are shown as flexed lines. The shaded rectangular boxes indicate the protein-coding regions. *Bcl6* mRNA was quantified using primer pair 1 in Fig. 3b and primer pair 2 in Supplementary Fig. 4g. (**g**) Quantitative RT-PCR analysis of the expression of *Bcl6* mRNA in naïve T cells stimulated with anti-CD3 and anti-CD28 for three days. The data were normalized relative to *Hprt* mRNA expression. (**h**) Autoradiography of SDS-PAGE gel to detect [^{35}S]-labeled proteins from nuclear or cytoplasmic extracts of T cells cultured for 3 days followed by metabolic labeling with [^{35}S]methionine/cysteine for 1 hr. Uncropped, full-size scans of autoradiogram shown in Fig. 3e. Data are representative of two independent experiments. NE; nuclear extract, CE; cytoplasmic extract.

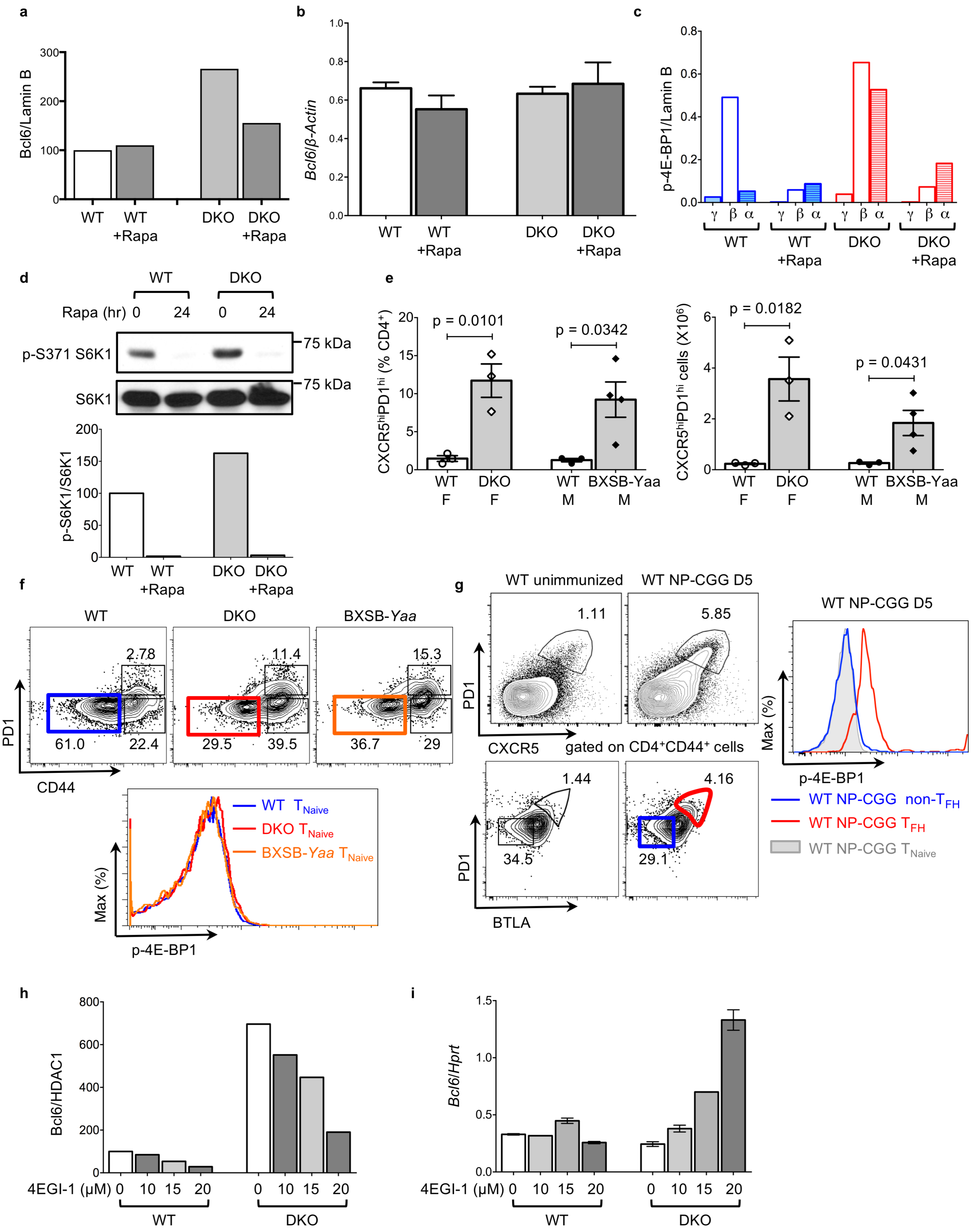
a
Bcl6/Lamin B
WT
WT +Rapa
DKO
DKO +Rapa
b
Bcl6/β-Actin
WT
WT +Rapa
DKO
DKO +Rapa
c
p-4E-BP1/Lamin B
γ β α
WT
WT +Rapa
DKO
DKO +Rapa
d
WT
DKO
Rapa (hr) 0 24 0 24
p-S371 S6K1
S6K1
75 kDa
75 kDa
p-S6K1/S6K1
WT
WT +Rapa
DKO
DKO +Rapa
e
p = 0.0101
p = 0.0342
CXCR5hiPD1hi (% CD4+)
WT F
DKO F
WT M
BXSB-Yaa M
p = 0.0182
p = 0.0431
CXCR5hiPD1hi cells (X10^6)
f
WT
DKO
BXSB-Yaa
PD1
CD44
2.78
61.0
22.4
11.4
29.5
39.5
15.3
36.7
29
WT TNaive
DKO TNaive
BXSB-Yaa TNaive
Max (%)
p-4E-BP1
g
WT unimmunized
WT NP-CGG D5
1.11
5.85
PD1
CXCR5
gated on CD4+CD44+ cells
1.44
34.5
4.16
29.1
BTLA
WT NP-CGG D5
Max (%)
p-4E-BP1
WT NP-CGG non-TFH
WT NP-CGG TFH
WT NP-CGG TNaive
h
Bcl6/HDAC1
4EGI-1 (μM) 0 10 15 20 0 10 15 20
WT
DKO
i
Bcl6/Hprt
4EGI-1 (μM) 0 10 15 20 0 10 15 20
WT
DKO

Supplementary Figure 5 | Analysis of *Bcl6* mRNA expression and mTORC1 targets. (**a**) The abundance of Bcl6 protein in the Western blots of Fig. 4a was quantified and normalized to Lamin B. The levels of Bcl6 in the wt control were set as 100%. (**b**) Quantitative RT-PCR analysis of the expression of *Bcl6* mRNA in sorted naïve wt or DKO T cells cultured as described in Figure 3. The data were normalized relative to *β-Actin* mRNA expression and are representative of two independent experiments. (**c**) The phosphorylation levels of 4E-BP proteins in Fig. 4b were quantified and normalized to Lamin B. The ratios of the levels of p-4E-BP to Lamin B are shown. (**d**) Western blot analysis of the levels of p-S6K1 in extracts from wt or DKO T cells. The phosphorylation levels of S6K1 protein were quantified and normalized to S6K1. The levels of p-S6K1 in the vehicle-treated wt control were set as 100%. (**e**) Quantification of the frequencies and numbers of T_{FH} cells ($CD4^{+}CXCR5^{hi}PD1^{hi}$) in wt, DKO, and BXSB-*Yaa* mice (n=3-4). Each dot represents an individual mouse. Error bars indicate mean ± s.d., p value by unpaired *t*-test. (**f**) Representative FACS plots for CD44 and PD1(top panel) or FACS histograms showing p-4E-BP expression (bottom panel) gated on naive T cells ($CD4^{+}CD44^{-}PD1^{-}$) in spleen from young (4-14-week-old) mice of the indicated genotype. Data are representative of at least two independent experiments. (**g**) Representative FACS plots for CXCR5 and PD1(top panel), BTLA and PD1 (bottom panel), or FACS histograms showing p-4E-BP expression (right panel) gated on indicated T cells in spleens from mice that were immunized 5 days earlier. Data are representative of two independent experiments. (**h**) The abundance of Bcl6 protein in the Western blots of Fig. 4f were quantified and normalized to HDAC1. Data are representative of two independent experiments. (**i**) Quantitative RT-PCR analysis of the expression of *Bcl6* mRNA in sorted naïve wt or DKO T cells cultured as indicated in Figure 3 and treated with vehicle control or the indicated dose of 4EGI-1 for the final 24 hours of the three-day culture. The data were normalized relative to *hprt* mRNA expression and are representative of two independent experiments.

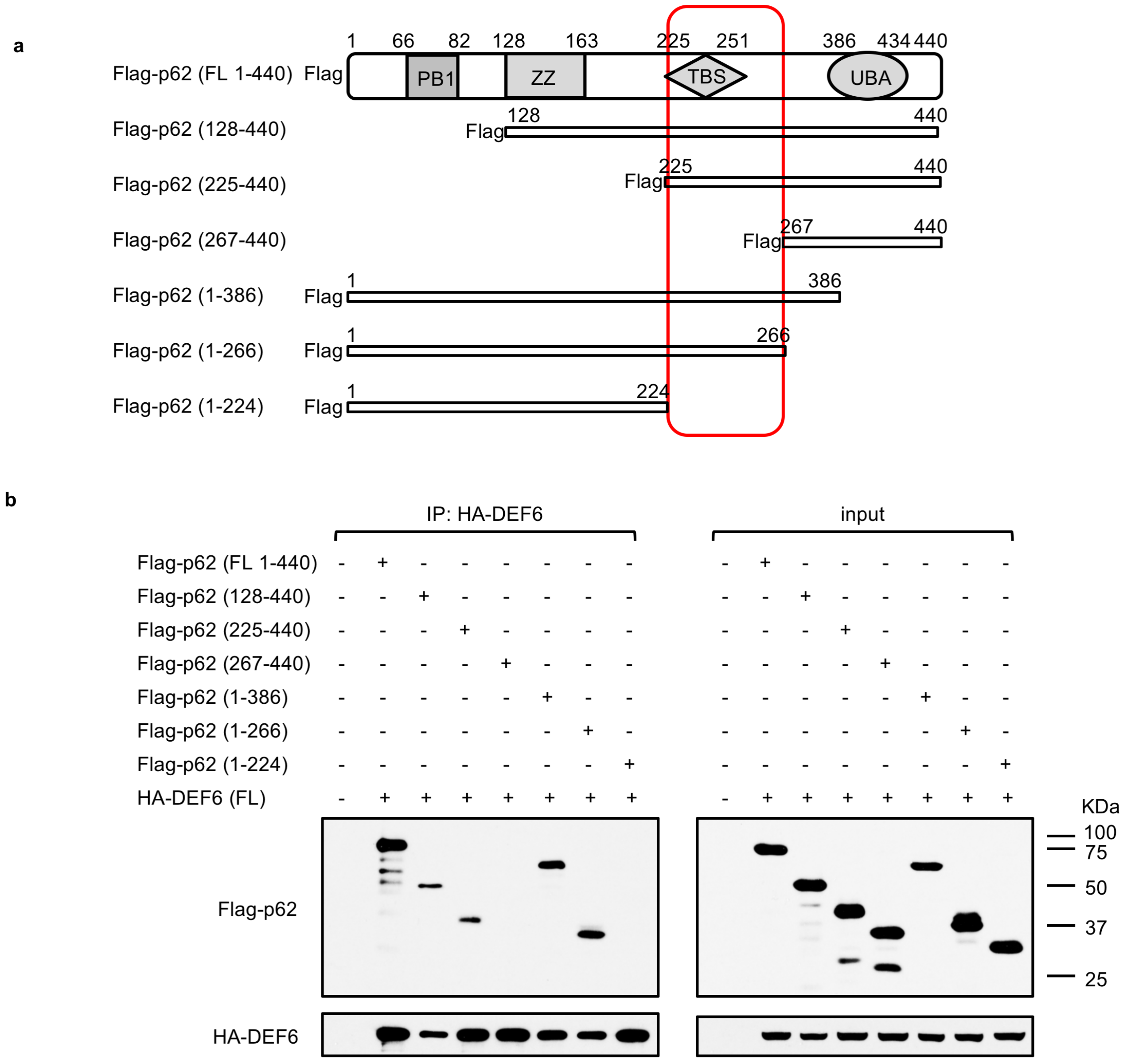

Supplementary Figure 6 | Mapping of the interaction of DEF6 with p62.
(**a**) Schematic representation showing protein interaction domains of SQSTM1/p62 and the deletion mutants utilized in the mapping experiments. PB1; a Phox and Bem1p domain, ZZ; an atypical zinc finger, TBS; TRAF6 binding sequence, UBA; a carboxy-terminal ubiquitin-associated domain. (**b**) 293T cells were cotransfected with HA-DEF6 (FL) and either Flag-p62 (FL 1-440), Flag-p62 (128-440), Flag-p62 (225-440), Flag-p62 (267-440), Flag-p62 (1-386), Flag-p62 (1-266), or Flag-p62 (1-224) expression constructs. HA-DEF6 immunoprecipitates were analyzed by Western blotting with the indicated antibodies to determine the interaction between DEF6 and various p62 deletion mutants. aa; amino acid, FL; full length.

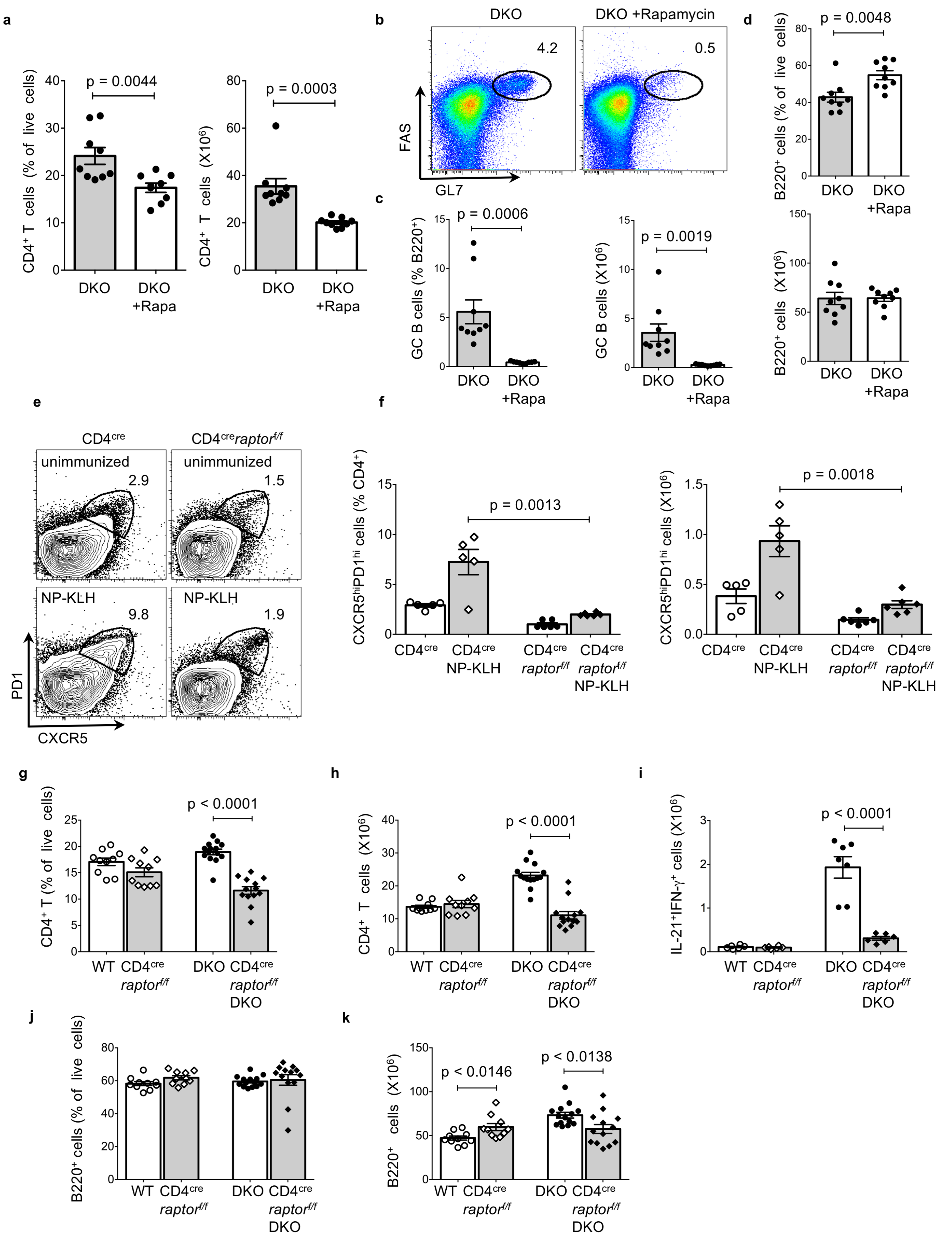
a
p = 0.0044
CD4+ T cells (% of live cells)
DKO
DKO +Rapa
p = 0.0003
CD4+ T cells (X10^6)
b
DKO
DKO +Rapamycin
4.2
0.5
FAS
GL7
c
p = 0.0006
GC B cells (% B220+)
p = 0.0019
GC B cells (X10^6)
d
p = 0.0048
B220+ cells (% of live cells)
B220+ cells (X10^6)
e
CD4cre
CD4cre raptor f/f
unimmunized
2.9
1.5
NP-KLH
9.8
1.9
PD1
CXCR5
f
p = 0.0013
CXCR5hiPD1hi cells (% CD4+)
p = 0.0018
CXCR5hiPD1hi cells (X10^6)
CD4cre NP-KLH
CD4cre raptor f/f
CD4cre raptor f/f NP-KLH
g
p < 0.0001
CD4+ T (% of live cells)
WT
DKO
CD4cre raptor f/f DKO
h
CD4+ T cells (X10^6)
i
IL-21+IFN-γ+ cells (X10^6)
j
B220+ cells (% of live cells)
k
p < 0.0146
p < 0.0138
B220+ cells (X10^6)

Supplementary Figure 7 | Regulation of DKO T_{FH} cells by mTORC1 *in vivo*. (**a**-**d**) Analysis of the spleens from aging (>35 week-old) DKO mice treated with vehicle control or rapamycin (3 mg kg^{-1}) daily for 10 days. Combined data from two independent experiments. (n=9 for the control mice and n=8 for rapamycin treated mice). (**a**) Quantification of $CD4^+$ T cells. (**b**) Representative FACS plots for GL7 and FAS gated on $B220^+$ cells. (**c**) Quantification of GC B cells ($GL7^+FAS^+B220^+$). (**d**) Quantifications of $B220^+$ B cells. (**e**) Representative FACS plots for CXCR5 and PD1 on $CD4^+$ T cells from $CD4^{cre}$ or $CD4^{cre}$*raptor*$^{f/f}$ mice that were either unimmunized (top panel) or immunized with NP-KLH in alum 7 days previously (bottom panel). The data are representative of two independent experiments (n=5-6). (**f**) Quantification of T_{FH} cells ($CD4^+CXCR5^{hi}PD1^{hi}$) (n=5-6) in $CD4^{cre}$ and $CD4^{cre}$*raptor*$^{f/f}$ mice. Mice were either unimmunized or immunized with NP-KLH in alum 7 days previously. Combined data from two independent experiments. Each dot represents an individual mouse. Error bars indicate mean ± s.d., p value by two-tailed *t*-test. (**g**,**h**) Quantification of $CD4^+$ T cells in the spleens of wt, $CD4^{cre}$*raptor*$^{f/f}$, DKO, and $CD4^{cre}$*raptor*$^{f/f}$DKO mice (n=10-14). Combined data from four independent experiments. Each dot represents an individual mouse. Error bars indicate mean ± s.d., p value by two-tailed *t*-test. (**i**) Quantification of $CD4^+$ T cells producing IFN-γ and IL-21 in the spleens of wt, $CD4^{cre}$*raptor*$^{f/f}$, DKO, and $CD4^{cre}$*raptor*$^{f/f}$DKO mice analyzed by intracellular flow cytometry. Data were combined from two independent experiments. (**j**,**k**) Quantification of $B220^+$ B cells in the spleens of wt, $CD4^{cre}$*raptor*$^{f/f}$, DKO, and $CD4^{cre}$*raptor*$^{f/f}$DKO mice (n=10-14). Combined data from four independent experiments.

a

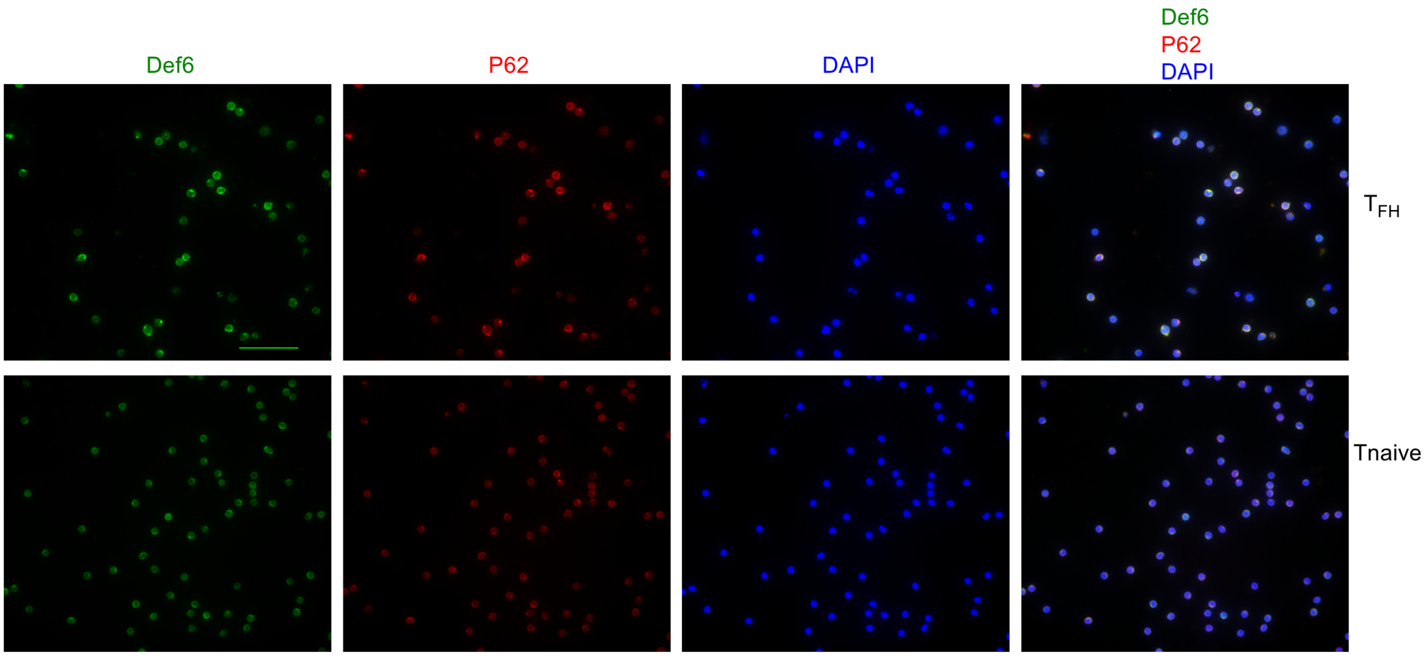

Supplementary Figure 8 | Localization of Def6 and P62. (**a**) Immunofluorescence images of Def6 and p62 in T_{FH} cell and naive T cells sorted from wt mice that were immunized with NP-CGG 7 days earlier. Representative images from two independent experiments. Def6, green; p62, red; DAPI, Blue. Scale bar, 50μm

Fig. 3a

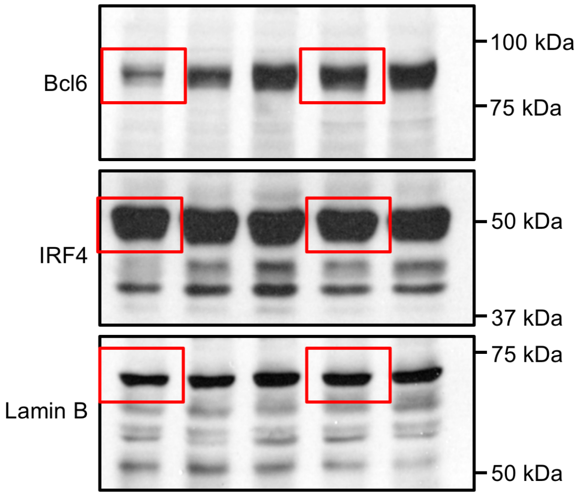

Fig. 3c

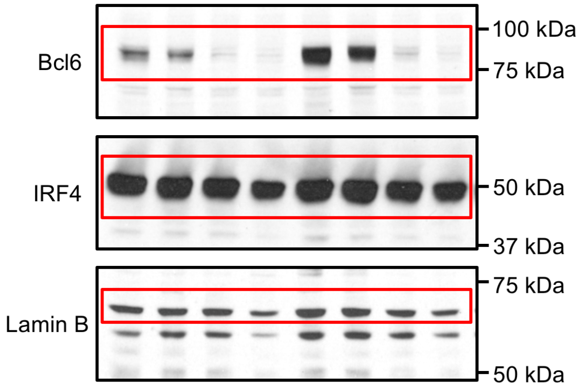

Fig. 4a

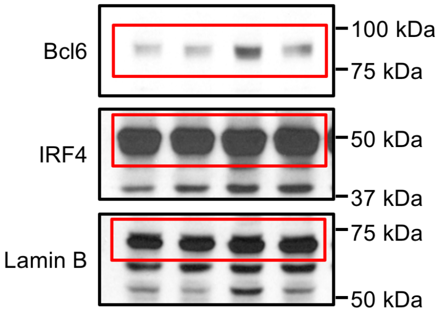

Fig. 4b

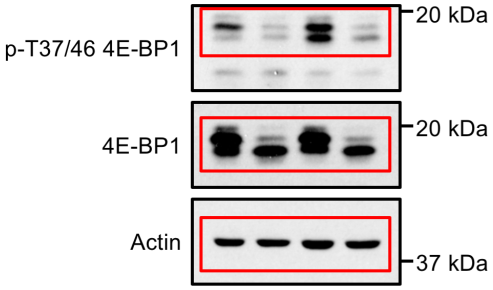

Fig. 4f

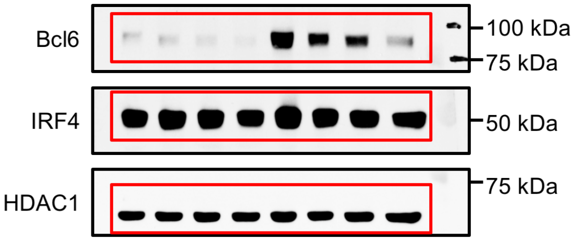

Supplementary Figure 9 | Uncropped images of the original scans of Western blots. Uncropped, full-size scans of immunoblots shown in Fig. 3a, Fig. 3c, Fig. 4a, Fig. 4b, and Fig. 4f.

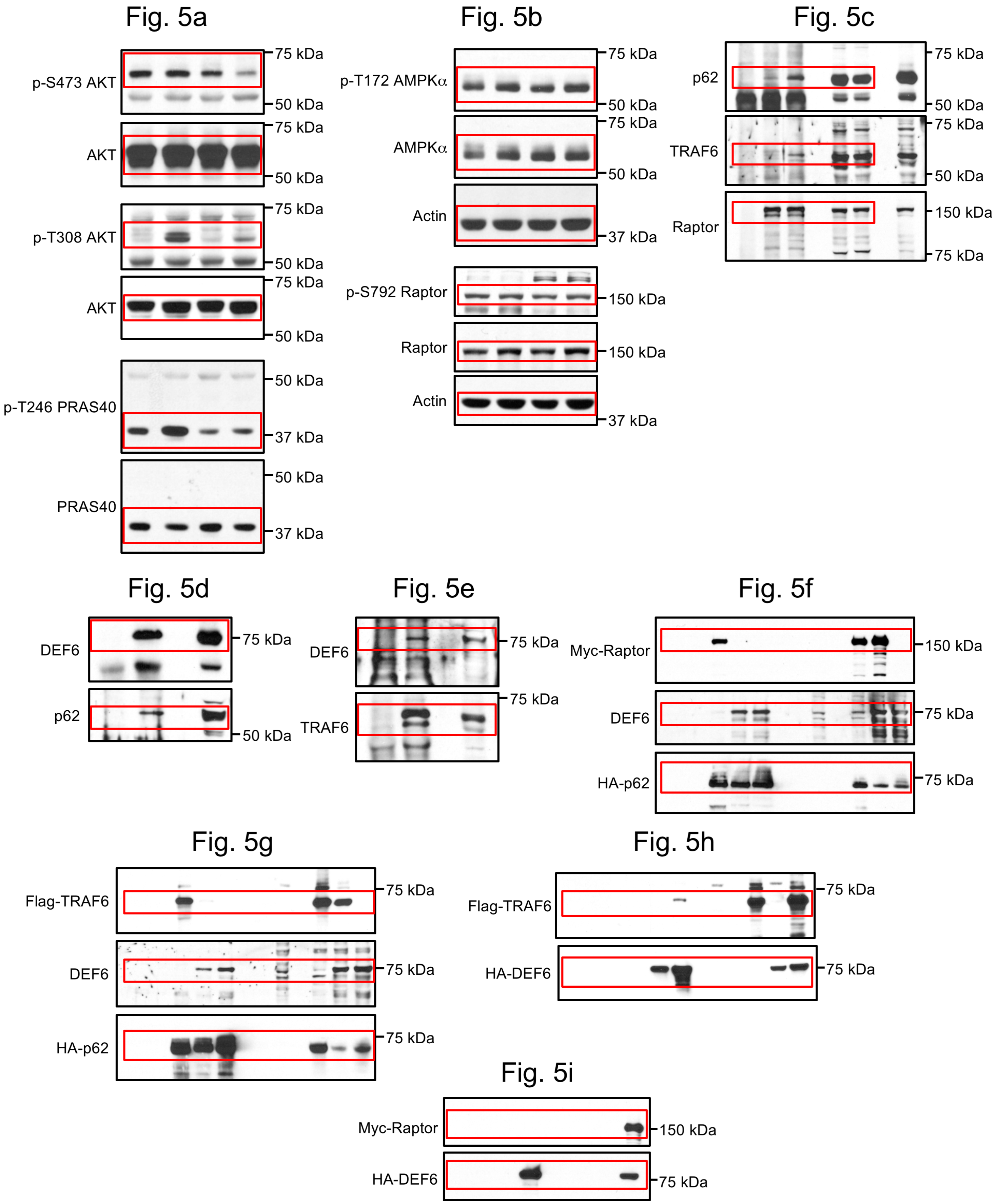

Supplementary Figure 10 | Uncropped images of the original scans of Western blots. Uncropped, full-size scans of immunoblots shown in Fig. 5a-i.

Fig. 6b

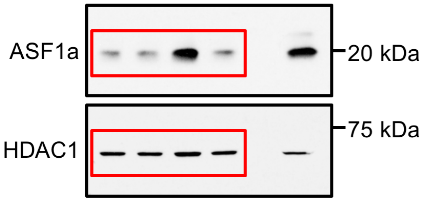

Fig. 6c

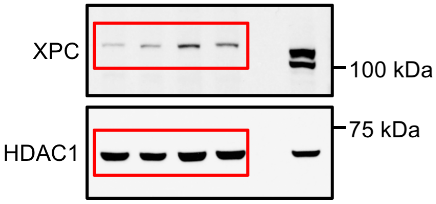

Supplementary Fig. 4c

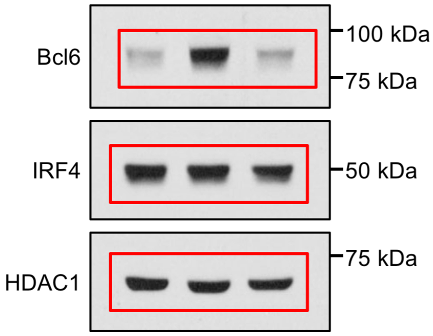

Supplementary Fig. 4d

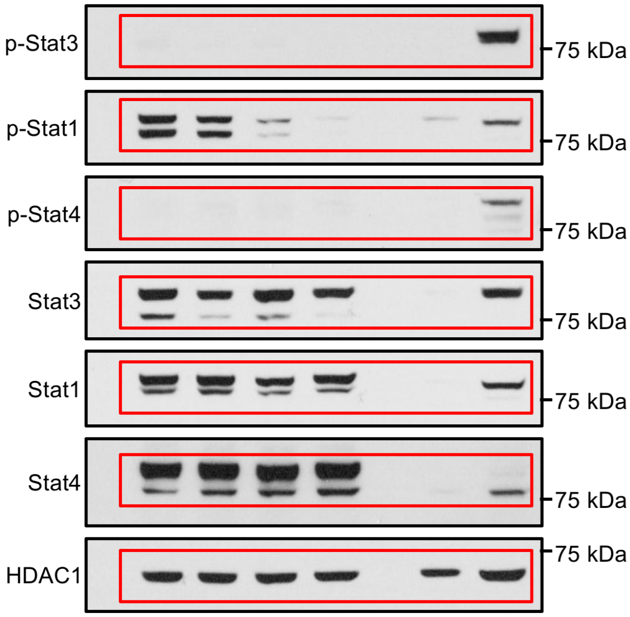

Supplementary Fig. 4e

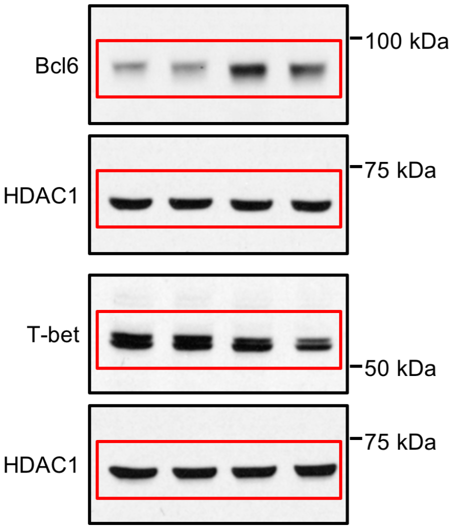

Supplementary Fig. 5d

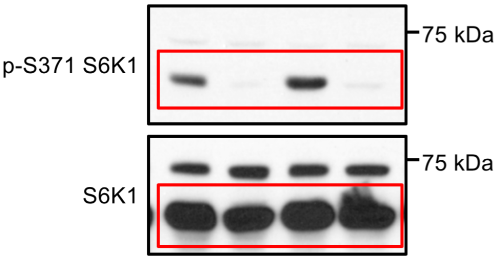

Supplementary Figure 11 | Uncropped images of the original scans of western blots. Uncropped, full-size scans of immunoblots shown in Fig. 6b, Fig. 6c, Supplementary Fig. 4c, Supplementary Fig. 4d, Supplementary Fig. 4e, and Supplementary Fig. 5d.
